# The Hog1 MAPK substrate governs *Candida glabrata*-epithelial cell adhesion via the histone H2A variant

**DOI:** 10.1371/journal.pgen.1011281

**Published:** 2024-05-14

**Authors:** Mahima Sagar Sahu, Rajaram Purushotham, Rupinder Kaur

**Affiliations:** 1 Laboratory of Fungal Pathogenesis, Centre for DNA Fingerprinting and Diagnostics, Hyderabad, India; 2 Graduate studies, Regional Centre for Biotechnology, Faridabad, Haryana, India; CAU: Christian-Albrechts-Universitat zu Kiel, GERMANY

## Abstract

CgHog1, terminal kinase of the high-osmolarity glycerol signalling pathway, orchestrates cellular response to multiple external stimuli including surplus-environmental iron in the human fungal pathogen *Candida glabrata* (*Cg*). However, CgHog1 substrates remain unidentified. Here, we show that CgHog1 adversely affects *Cg* adherence to host stomach and kidney epithelial cells in vitro, but promotes *Cg* survival in the iron-rich gastrointestinal tract niche. Further, CgHog1 interactome and in vitro phosphorylation analysis revealed CgSub2 (putative RNA helicase) to be a CgHog1 substrate, with CgSub2 also governing iron homeostasis and host adhesion. CgSub2 positively regulated *EPA1* (encodes a major adhesin) expression and host adherence via its interactor CgHtz1 (histone H2A variant). Notably, both CgHog1 and surplus environmental iron had a negative impact on CgSub2-CgHtz1 interaction, with *CgHTZ1* or *CgSUB2* deletion reversing the elevated adherence of *Cghog1Δ* to epithelial cells. Finally, the surplus-extracellular iron led to CgHog1 activation, increased CgSub2 phosphorylation, elevated CgSub2-CgHta (canonical histone H2A) interaction, and *EPA1* transcriptional activation, thereby underscoring the iron-responsive, CgHog1-induced exchange of histone partners of CgSub2. Altogether, our work mechanistically defines how CgHog1 couples Epa1 adhesin expression with iron abundance, and point towards specific chromatin composition modification programs that probably aid fungal pathogens align their adherence to iron-rich (gut) and iron-poor (blood) host niches.

## Author summary

The human gut can serve as a source of disseminated infections caused by its resident microbes. *Candida glabrata* (*Cg*) is one such opportunistic fungal pathogen that is isolated from both iron-rich (human gut) and iron-poor (blood) host niches. *Cg* is presumed to translocate across the epithelial cell barrier of the gastrointestinal tract into the bloodstream. However, how *Cg* colonizes and traverses through the host gut, and adapts to the iron-rich gut milieu, is unknown. We show that *Cg* ingeniously attunes expression of the major cell surface protein Epa1, which mediates adherence to the host epithelial cells, to the environmental iron abundance, by exchanging the interacting partner (histone variant CgHtz1) of the CgSub2 RNA helicase. We also show that the conserved mitogen-activated protein kinase CgHog1, that may control this partner exchange by phosphorylating CgSub2, is essential for *Cg* survival in the mouse gut and bloodstream infections. Altogether, our work yields molecular insights into how nutrient fluctuations in the mammalian host lead to the chromatin-based transcriptional rewiring of a key virulence gene.

## Introduction

Transcriptional regulatory networks fine-tune gene expression in response to environmental cues, and are pivotal to stress adaptation and fungal virulence [1,2]. Iron is an essential element whose abundance fluctuates in the human host, with blood and gut representing iron-poor and iron-rich niches, respectively [3]. Fungal pathogens employ mitogen-activated protein kinase (MAPK) signalling pathways to transmit the extracellular nutrient signal and generate an apt intracellular response via the reprogrammed gene expression [1,2]. The MAPK signalling module consists of three kinases that are sequentially activated by dual phosphorylation of the conserved threonine and tyrosine residues in the TXY motif, with the terminal kinase often activating the environmental stress-cognate transcription factor [1,2]. CgHog1, terminal kinase of the high osmolarity glycerol (HOG) MAPK signalling cascade, and an ortholog of the mammalian p38 MAPK, responds to iron availability in the human opportunistic fungal pathogen *Candida glabrata* [2,4].

*C*. *glabrata* (*Cg*), also known as *Nakaseomyces glabrata*, cohabits microbiota in the oral cavity, gastrointestinal and vaginal tracts in healthy individuals, but causes mucosal and life-threatening invasive infections in immunocompromised patients [5–7]. Based on the geographical region, *Cg* ranks second to fourth among the most frequently isolated *Candida* species in bloodstream infections, with *Cg* infections accounting for < 35% mortality [8–13]. The World Health Organization has classified *Cg* as a high-priority fungal pathogen [14]. Although elevated resistance towards different stresses contribute to *Cg* pathogenesis [7], mechanisms regulating environmental stress responses in *Cg* remain elusive.

The HOG MAPK pathway is essential for thermal, osmotic, oxidative and high-iron stress survival [4]. CgHog1 also aids *Cg* proliferate in macrophages, compete with *Lactobacillus spp*. in the vaginal mucosa, and survive in the mouse systemic candidiasis model [4,15]. Iron limitation, iron excess and endoplasmic reticulum stresses activate CgHog1 [4,16]. Further, CgHog1 kinase regulates the expression of one-quarter of *Cg* genes, with *CgHOG1* deletion repressing and inducing 426 and 831 genes, respectively [4]. *CgHOG1* loss also results in high intracellular iron content, downregulation of the high-affinity iron uptake genes, increased expression of the major cell wall adhesin-encoding gene *EPA1*, and elevated adhesion to Lec2 epithelial cells [4].

*Cg* is overrepresented in inflammatory bowel disease patients [17], and presumed to translocate across the epithelial cell barrier of the gastrointestinal tract into the bloodstream [18–20]. However, mechanisms that facilitate this spread remain unknown. The gastrointestinal tract is an iron-rich host niche, primarily due to non/partial absorption of dietary iron, though few parts within this niche may be iron-restricted [3,21,22]. In *Cg*, the high environmental iron de-represses cell surface adhesin expression by relieving subtelomeric gene silencing, and leads to elevated adherence to host epithelial cells and increased biofilm formation in vitro [4,23]. Elevated adherence may facilitate *Candida* colonization and persistence during commensalism in the gastrointestinal tract [20,24]. Notably, *Cg* evoked adhesin-specific immunoglobulin A response in the gut, with IgA maintaining intestinal homeostasis and promoting commensalism [25].

CgHog1 regulates iron homeostasis and epithelial cell adherence [4]. However, we know nothing about the molecular players that help CgHog1 govern host adhesion in an iron-rich environment and promote *Cg* pathogenesis. By identifying CgHog1 interactors in varied-iron conditions, we, here, have expanded the functional repertoire of CgHog1, and established a putative RNA helicase CgSub2 and the histone H2A variant CgHtz1 as two new components of the iron response and host adhesion machinery in *Cg*. Additionally, we demonstrate that CgHog1 phosphorylates CgSub2, and that CgHog1-mediated negative regulation of CgSub2-CgHtz1 interaction governs host adhesion in an iron-rich milieu.

## Results

### CgHog1 is required for *Cg* survival in the gastrointestinal candidiasis model

**Fig 1:**
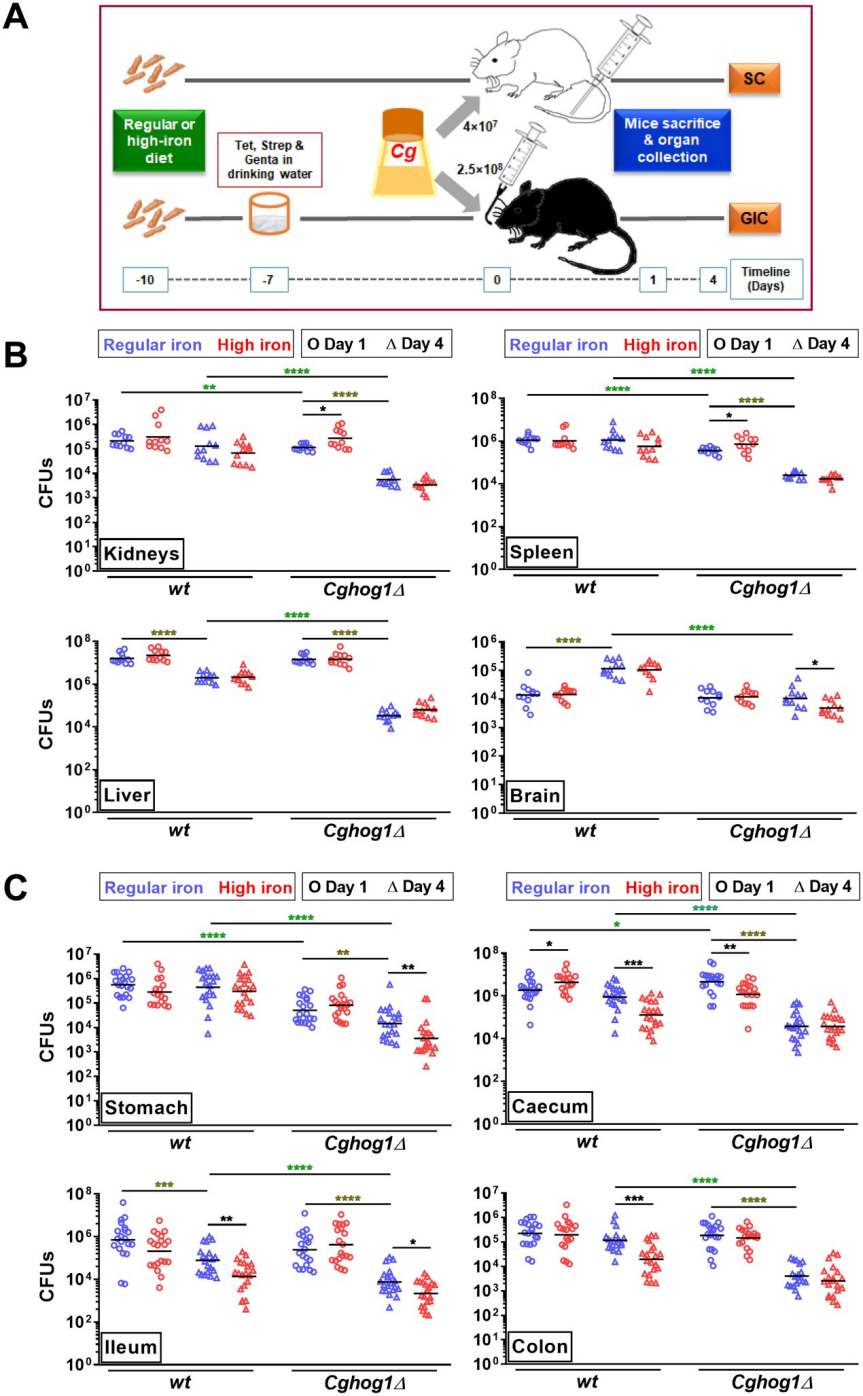
Iron abundance impacts *Cg* colonization of the gastrointestinal tract. **A**. **Schematic diagram of the experimental design for systemic candidiasis (SC) and gastrointestinal candidiasis (GIC)**. **B** and **C**. Groups of regular-iron (190 mg/kg diet) or high-iron (500 mg/kg diet) diet-fed BALB/c (B) and C57BL/6 (C) mice were infected with *C*. *glabrata* (*Cg*) via tail-vein injection and oral gavage, respectively. At 1^st^ and 4^th^ day post-infection, mice were sacrificed and fungal load in indicated organs was determined. **wild-type (*wt;* YRK20) and *Cghog1Δ* (YRK964) survival in SC (B;** n = 11**) and GIC (C;** n = 17–20**) models.** Circles and triangles represent CFUs in individual mouse organs at 1^st^ and 4^th^ day post-infection, respectively. Bars indicate the CFU geometric mean. Black asterisks denote differences in organ CFUs between regular and high iron-fed mice infected with the same *C*. *glabrata* strain and sacrificed on the same day. Green asterisks denote organ CFU differences between regular iron-fed and same day-sacrificed *wt-* and *Cghog1Δ*-infected mice. Olive asterisks denote organ CFU differences between regular iron-fed, same *C*. *glabrata* strain-infected 1^st^ and 4^th^ day-sacrificed mice. *, p ≤ 0.05; **, p ≤ 0.01; ***, p ≤ 0.001; ****, p ≤ 0.0001, Mann-Whitney U-test.

*Cg* resides in the iron-rich gastrointestinal tract, and CgHog1 is essential for survival of the high-iron stress in vitro and for *Cg* virulence in the murine model of systemic candidiasis [4,18,20,26]. However, how iron abundance modulates *Cg* survival in systemic and gastrointestinal candidiasis models is unknown. To address this, we examined wild-type (*wt*) and *Cghog1Δ* survival in both models wherein the mice were fed either a regular-iron (RI; 190 mg/kg diet) or a high-iron (HI; 500 mg/kg diet) diet for 10 days, prior to infection (Fig 1A). In the systemic candidiasis model, we recovered similar *wt* CFUs (colony-forming units) from the kidneys, liver, spleen and brain of RI diet- and HI diet-fed mice at day 1 (d1) and day 4 (d4) post-infection (Fig 1B). These results suggest that the surplus-iron does not impact *wt* survival appreciably during the early infection stages in the systemic model. Notably, *wt* d4-CFUs in the brain were 7-fold higher than d1-CFUs (Fig 1B), indicative of *Cg* proliferation in the brain. *Cghog1Δ* d4- brain CFUs were 2.5-fold lower in HI diet-fed mice, compared to RI diet-fed mice (Fig 1B), indicating that surplus iron may impair *Cghog1Δ* growth in vivo (Fig 1B). Importantly, compared to *wt* 4d-CFUs, diminished 4d-CFUs of *Cghog1Δ* in all organs, kidneys, liver, spleen and brain (Fig 1B), corroborate the earlier finding of CgHog1 being required for *Cg* survival in the murine systemic candidiasis model [4]. Altogether, these results suggest that increased iron abundance does not promote *Cg* survival in the systemic model of candidiasis.

Next, we examined *Cg* burden in the gastrointestinal candidiasis model. D1-CFUs for *wt* and *Cghog1Δ* were 2- and 4-fold higher and lower, respectively, in the caecum of HI diet-fed mice, compared to respective RI diet-fed mice (Fig 1C), suggesting that while high iron aids *wt* colonization of the mouse caecum, it is detrimental to *Cghog1Δ* survival. Notably, the caecal-d4- *wt* CFUs were lower in HI diet-fed mice, compared to RI diet-fed mice (Fig 1C), indicating that high-iron negatively impacts *wt* survival, and that, d1- and d4-CFUs may represent initial colonization and survival in tissues, respectively. Since *Cghog1Δ* d1-CFUs in RI-fed mice ceacum were 2.5-fold higher than *wt* d1-CFUs, it is conceivable that *Cghog1Δ* colonizes caecum better at day 1 (Fig 1C). Importantly, compared to *wt*, *Cghog1Δ* d4-CFUs were lower in all organs, stomach, ileum, caecum and colon (Fig 1C), thereby reinforcing CgHog1 functions in promoting *Cg* survival in the host.

The *Cghog1Δ* mutant grows slowly, is sensitive to high-iron stress in vitro (4), and exhibited 1.3- and 3.0-fold lower cell density, compared to *wt*, after 24 to 48 h growth in YPD and YNB medium, respectively (S1A Fig). Thus, we infected RI diet-fed mice with a 3-fold higher *Cghog1Δ* inoculum to investigate the effect of mutant’s reduced growth on organ colonization and its survival in the gastrointestinal candidiasis model. We found similar results with the increased inoculum, with *Cghog1Δ* displaying reduced survival in the stomach at d1 and d4-post infection, and in the caecum, colon and ileum at d4-post-infection, compared to *wt* (S1B Fig). Notably, *Cghog1Δ* d1-CFUs in the caecum were higher than *wt* d1-CFUs (S1B Fig), indicating that CgHog1 adversely affects the initial *Cg* colonization of the caecum. Importantly, *Cghog1Δ*, at both 1X and 3X inoculum, failed to survive as well as the *wt* strain at 4^th^ day post-infection (S1B Fig), implicating CgHog1 in *Cg* survival in the mouse gastrointestinal candidiasis model. Altogether, while underscoring CgHog1 essentiality for *Cg* survival in both systemic and gastrointestinal models, these results suggest that the increased iron abundance may confer a transient advantage to *Cg* for colonization in the caecum, but it has no long-term positive impact on *Cg* survival in the gastrointestinal candidiasis model. Notably, Hog1 and Sfu1 (a negative regulator of iron-uptake genes in the gut) in *C*. *albicans* are required for mouse gut colonization and persistence, respectively [27,28], with surplus iron and *HOG1* deletion in *C*. *albicans* resulting in cell flocculation and perturbed iron homeostasis, respectively [29]. Further, intestinal expansion and translocation of *C*. *albicans* and *C*. *parapsilosis* into the bloodstream has been reported in transplant patients [30], thereby linking intestinal survival with *Candida* bloodstream infections. Collectively, our findings highlight CgHog1 requirement for *Cg* survival in the gastrointestinal candidiasis model.

### Identification of CgHog1 interactome

**Fig 2:**
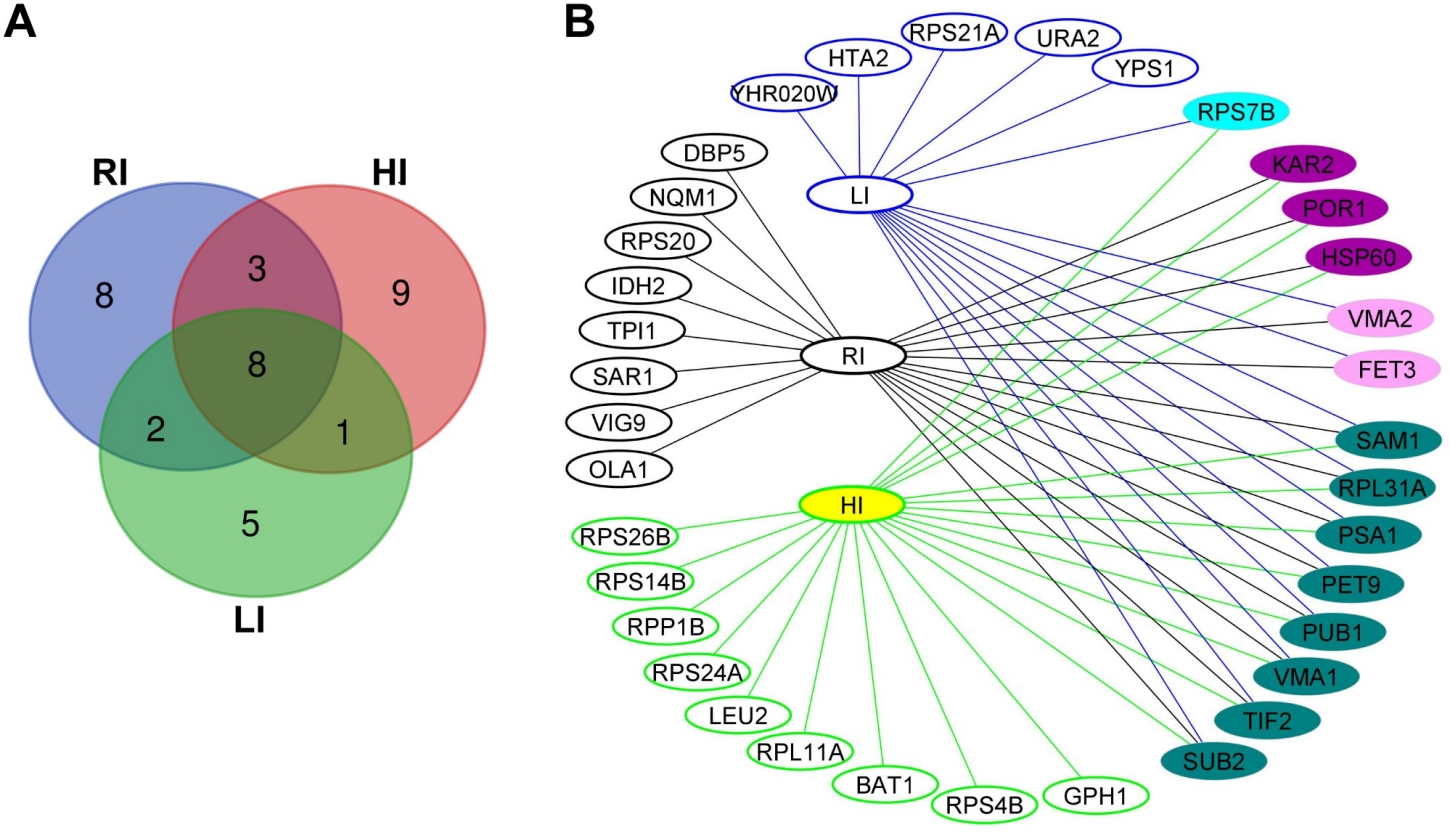
CgHog1 interactome analysis. **A. Venn diagram illustrating the overlap among CgHog1 interactors that were identified under low (LI), regular (RI), and high-iron (HI) conditions. B. Cytoscape network illustrating unique and common interactors of CgHog1 under LI, RI and HI conditions.** Blue, black and green-colored circles represent unique interactors of CgHog1 under LI, RI and HI conditions, respectively. Cyan, violet and pink color-filled circles represent common interactors of CgHog1 under LI and HI, RI and HI, and RI and LI, respectively. Dark green color-filled circles represent the common interactors of CgHog1 under all three-iron conditions.

To examine how CgHog1 regulates iron homoeostasis and *Cg* survival in the host, we next identified CgHog1 interactors, using the N-terminally SFB-tagged CgHog1, whose functionality was verified by rescue of osmotic stress and surplus-iron sensitivity of *Cghog1Δ* (S2A Fig), and by activation upon growth in the iron-rich medium (S2B Fig). We found 16, 21 and 21 proteins to interact with CgHog1 under growth in low [CAA containing extracellular iron chelator BPS (bathophenanthroline disulphonate)], regular (CAA), and high (CAA containing sodium ascorbate and ferrous ammonium sulfate)-iron medium, respectively (Fig 2A and S1 Table). These three datasets shared 8 common proteins (Fig 2A and 2B), which belonged to ‘Heme transport’, ‘ATP metabolic process’, ‘Cellular response to hypoxia’, ‘Ion transmembrane transport’ and ‘Regulation of mRNA stability’ gene ontology (GO) terms for Biological Process (BP), as revealed by FungiFun analysis (S2 Table). ‘Translation’ and ‘mRNA export from nucleus’ GO terms were uniquely enriched in the CgHog1 interactome under high- and regular-iron conditions, respectively (S2 Table). The core histone H2A was present in the low-iron interactome, while the high-affinity iron uptake system component, CgFet3 ferroxidase, was identified in both low- and regular-iron interactomes of CgHog1 (Fig 2B and S1 Table). Notably, three common CgHog1 interactors between regular- and high-iron grown cells were CgHsp60 (mitochondrial chaperonin), CgKar2 (ATPase involved in protein import into the Endoplasmic Reticulum) and CgPor1 (mitochondrial voltage-dependent anion channel) that are involved in ATP-dependent protein folding, unfolded protein response and protein folding in the ER, and mitochondrial osmotic stability and membrane permeability, respectively (S1 Table). Altogether, these results suggest that CgHog1 interacts with more functionally-diverse proteins than previously anticipated.

### CgSub2, a CgHog1 interactor, is essential for cell viability

From identified CgHog1 interactors, we selected CgSub2 for further analysis for five reasons. First, it is neither characterized in *Cg* nor implicated in Hog1 signalling in any other fungal pathogen. Second, CgSub2 interacted with CgHog1 under all three (low, regular and high)-iron conditions (S1 Table). Third, Sub2 in *Saccharomyces cerevisiae* is a transcription export complex subunit, and a DEAD-box RNA helicase, that is involved in nuclear mRNA export and spliceosome assembly [31–35]. Fourth, *SUB2* overexpression upregulates iron homeostasis genes and suppresses heterochromatic gene silencing at telomeres in *S*. *cerevisiae* [36]. Finally, both *CgHOG1* deletion and surplus environmental iron in *Cg* activates expression of the subtelomerically-encoded *EPA1* gene [4].

**Fig 3:**
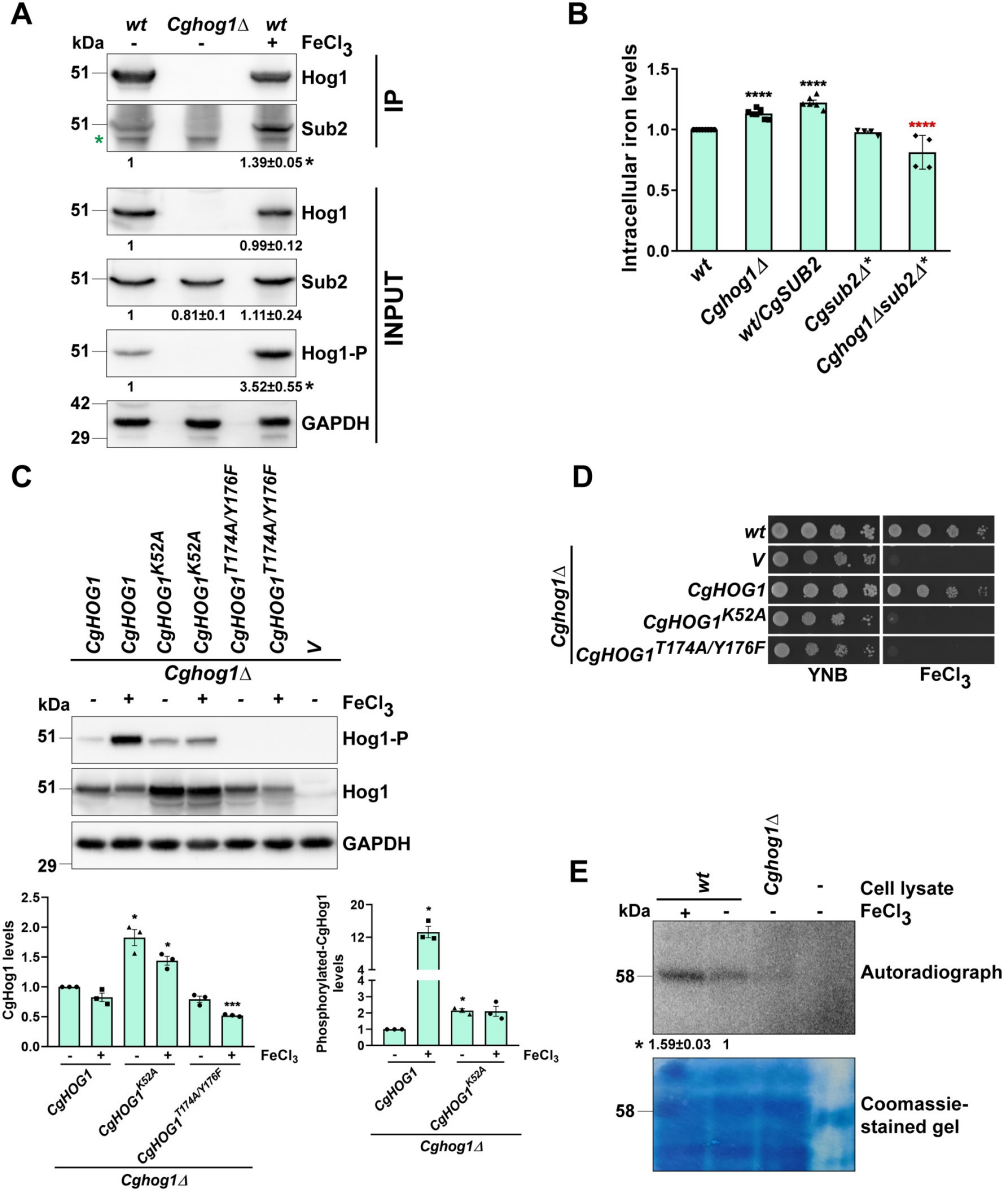
Surplus-iron increases CgHog1-mediated phosphorylation of CgSub2. **A. A representative Western blot showing elevated CgHog1-CgSub2 interaction in *wt* (YRK20) grown in ferric chloride (1 mM)-containing YNB medium for 2 h.** Cell lysates were immunoprecipitated (IP) using anti-CgHog1 antibody, and resolved on 10% SDS-PAGE. *Cghog1Δ* (YRK964) was used as negative control. For input samples, 100, 100, 120 and 60 μg protein were loaded for CgSub2, CgHog1, phohphorylated-CgHog1 (Hog1-P) and CgGapdh detection, respectively. CgGapdh was used as loading control. The signal intensity in each lane was quantified using the ImageJ software, and CgSub2 intensity values in input samples were normalized against the corresponding CgGapdh signal values, followed by normalization of the immunoprecipitated CgSub2 against the pulled-down, CgGapdh-normalized CgHog1 signal. Fold-increase (mean ± SEM, n = 3) in CgHog1-CgSub2 interaction in high-iron-grown *wt* cells, compared to regular-iron (YNB)-grown *wt* cells (considered as 1.0), is shown underneath the blot. *, *p* ≤ 0.05, paired two-tailed Student’s t-test. The green asterisk marks non-specific band. IP, Immunoprecipitated samples. **B. Intracellular iron levels in indicated strains.** *wt* (YRK20), *Cghog1Δ* (YRK964), and *wt/CgSUB2* (YRK2803) strains were grown to log-phase in YNB medium, while Cgsub2Δ* (YRK3294) and Cghog1Δsub2Δ* (YRK3372) strains were grown in methionine (2 mM) and cysteine (2 mM)-containing YNB medium. Data represent mean ± SEM (n = 4–8). Black and red asterisks denote fold-differences in iron content between indicated strains and *wt* (taken as 1.0), and between *Cghog1Δ* and *Cghog1Δsub2Δ*, respectively. ****, p ≤ 0.0001; two-tailed Student’s *t* test. **C. A representative Western blot showing CgHog1 phosphorylation (CgHog1-P) in CgHog1 variants-expressing *Cghog1Δ* that was grown in YNB medium lacking or containing 1 mM ferric chloride for 2 h.** CgGapdh was used as loading control. The signal intensity in each lane was quantified using the ImageJ software, and CgHog1 and CgHog1-phosphorylation signal intensity values in input samples were normalized against the corresponding CgGapdh signal values, followed by normalization of the phosphorylated-CgHog1 against the total CgHog1 protein signal. Fold-difference in CgHog1 and CgHog1-phosphorylation levels (mean ± SEM, n = 3) in indicated condition/strains is shown, compared to regular-iron (YNB)-grown *Cghog1Δ/CgHOG1* cells (considered as 1.0). *, *p* ≤ 0.05; ***, p ≤ 0.001, paired two-tailed Student’s t-test. *Cghog1Δ+CgHOG1*, *Cghog1Δ+CgHOG1*^*K52A*^, *Cghog1Δ+CgHOG1*^*T174A/Y176F*^ and *Cghog1Δ/V* strains correspond to YRK1047, YRK2127, YRK2129 and YRK1166 strains, respectively. **D. Serial dilution spotting assay.** Overnight YPD medium-grown cultures were normalized to OD_600_ of 1.0, followed by spotting of 3 μl of three 10-fold serial dilutions on YNB medium lacking or containing 3 mM FeCl_3_. V, empty vector. **E. A representative autoradiograph showing recombinant CgSub2 (rCgSub2) phosphorylation.** Log-phase *wt* (YRK20) cultures were either treated with 1 mM FeCl_3_ for 2 h or left untreated in YNB medium, while *Cghog1Δ* (YRK964) cultures were kept untreated. After cell lysis, whole cell lysates (200 μg) were incubated with 100 μg *E*. *coli-*purified rCgSub2, 0.5 mM ATP and 10 μCi γ-^32^P-ATP at 30°C for 1 h. As a negative control, rCgSub2 was incubated with 0.5 mM ATP and 10 μCi γ-^32^P-ATP without *Cg* cell lysate. Samples were resolved on 10% SDS-PAGE and analyzed by autoradiography. The last lane contained all reagents but for the cell lysate. The signal intensity in lanes was quantified using the ImageJ software, and fold-increase in CgSub2 phosphorylation (mean ± SD, n = 2) is shown in high-iron-grown *wt*, compared to regular-iron-grown *wt* cells (considered as 1.0). *, *p* ≤ 0.05, paired two-tailed Student’s t-test.

To verify and characterize CgHog1-CgSub2 interaction, we first generated antibodies against *Escherichia coli*-purified CgSub2 and CgHog1 proteins in mice, and checked their specificities (S3A and S3B Fig). Next, we verified the interaction between endogenous CgSub2 and CgHog1 proteins, and found increased CgSub2-CgHog1 interaction under high-iron condition (Fig 3A). Notably, high-iron had no appreciable effect on CgSub2 and CgHog1 protein levels, but it activated CgHog1, as evidenced in elevated CgHog1 phosphorylation (Fig 3A). CgSub2 also interacted with the phosphorylated form of CgHog1 (S3C Fig), further corroborating CgHog1-CgSub2 interaction.

To study CgSub2 functions in *Cg*, we sought to generate a *CgSUB2* deletion strain. However, our attempts to delete *CgSUB2* gene were unsuccessful, indicating that it may be essential for *Cg* growth. Therefore, we generated a conditional *CgSUB2*-deletion strain, that expressed *CgSUB2* from methionine (met)-repressible *CgMET3* promoter. This *Cgsub2Δ/MET3Pro-CgSUB2* strain will be referred to as Cgsub2Δ* from hereon, and reflects the conditional deletion of *CgSUB2*. Cgsub2Δ* grew poorly on both methionine and cysteine (cys)-containing solid (S3D Fig) and liquid medium (S3E Fig), with cellular CgSub2 levels decreasing gradually over time in the medium containing methionine and cysteine (S3F Fig).

Next, we checked the effect of CgSub2 loss on iron homeostasis, as CgHog1 is pivotal to maintain intracellular iron content, with *Cghog1Δ* containing elevated iron levels [4]. We found that while *CgSUB2* conditional deletion had no effect on the intracellular iron levels in *wt*, it led to a reversal of the high intracellular iron content of *Cghog1Δ* (Fig 3B), thereby implicating CgSub2 in perturbed iron homeostasis of *Cghog1Δ*. As a control, we also checked if methionine and cysteine addition to the medium alters intracellular iron levels. We found similar iron levels in *wt* cells that were grown in the medium lacking or containing methionine and cysteine (S3G Fig). Similarly, methionine and cysteine presence in the medium had no effect on elevated iron levels in the *Cghog1Δ* mutant (S3G Fig).

Further, to check the effect of *CgSUB2* overexpression, we expressed *CgSUB2* in *wt* cells from the strong *PDC1* promoter, and found substantially higher CgSub2 protein levels in *CgSUB2*-overexpressing strain (*wt/CgSUB2*), compared to the *wt* strain containing endogenous CgSub2 (S3H Fig). Notably, we found that *CgSUB2* overexpression led to increased intracellular iron levels in *wt* (Fig 3B), indicating that CgSub2 may govern cellular iron content maintenance in *Cg*. Altogether, these data suggest that like CgHog1, its interactor CgSub2 also regulates iron homeostasis, and that, elevated iron in *Cghog1Δ* could be due to altered CgSub2 functions.

### CgHog1 phosphorylates CgSub2

Next, to investigate if CgSub2 is a substrate of CgHog1, we performed two experiments. First, we identified catalytic and phosphorylated amino acid residues in CgHog1 through multiple sequence alignment of Hog1 of human and yeast species (S4A Fig). Of note, Hog1 is a conserved MAPK and p38 is the human homolog of CgHog1, with MAP kinase kinases activating p38 MAPKs by phosphorylating Threonine and Tyrosine in the dual phosphorylation motif, Thr-X-Tyr [1,2]. Our in silico analysis predicted Lysine-52, and Threonine-174 and Tyrosine-176, to be required for CgHog1 kinase activity and HOG pathway activation, respectively (S4A Fig). Thus, we simultaneously mutated the Thr-174 and Tyr-176 residues in CgHog1 to alanine and phenylalanine, respectively, to generate the non-phosphorylatable CgHog1. Additionally, Lys-52 was mutated to alanine to generate the catalytically-inactive CgHog1. We found that Lys-52 mutation led to higher protein levels, and diminished high iron-induced increase in CgHog1 phosphorylation (Fig 3C). Contrarily, both basal and high-iron-responsive CgHog1 phosphorylation were absent in *Cghog1Δ*-expressing CgHog1^*T174A/Y176F*^ (Fig 3C), indicating that Thr-174 and Tyr-176 are phosphorylatable residues, that undergo phosphorylation upon HOG pathway activation. Notably, neither CgHog1^*K52A*^ nor CgHog1^*T174A/Y176F*^ could rescue the high-iron sensitivity of *Cghog1Δ* (Fig 3D), suggesting that the activated HOG pathway and CgHog1 activity are pivotal to high-iron stress survival.

Second, we purified CgSub2 from *E*. *coli* for *in vitro* phosphorylation assay. Co-incubation of 6XHis-tagged CgSub2 and γ-^32^P-ATP with whole cell lysates of *wt*, grown in regular- and high-iron medium, revealed the phosphorylated CgSub2 in both lysates, with CgSub2 phosphorylation signal being significantly higher in high-iron samples (Fig 3E). Notably, CgSub2 was not autophosphorylated, and CgSub2 phosphorylation was significantly reduced in the absence of CgHog1 (Fig 3E). A faint signal in *Cghog1Δ* (Fig 3E) may indicate that another kinase is capable of CgSub2 phosphorylation. Further, we also verified high-iron induced increase in CgSub2 phosphorylation by performing *in vitro* phosphorylation assay using cold ATP, and running samples on a Phos-tag gel. A clear mobility shift of CgSub2 was observed with cell lysates of high-iron grown *wt*, compared to regular-iron-grown *wt* (S4B Fig), indicating that CgSub2 is phosphorylated under excess-iron conditions. Importantly, both HOG pathway and CgHog1 kinase activity are required for CgSub2 phosphorylation, as *Cghog1Δ*-expressing catalytically-dead CgHog1^*Lys52A*^ or non-activatable CgHog1^*T174A/Y176F*^ could not phosphorylate CgSub2 (S4C Fig). Collectively, these results suggest that CgHog1 phosphorylates CgSub2, with surplus-iron-inducible HOG pathway activation elevating the CgHog1 kinase activity.

### CgSub2 modulates *Cg* adherence to host epithelial cells

**Fig 4:**
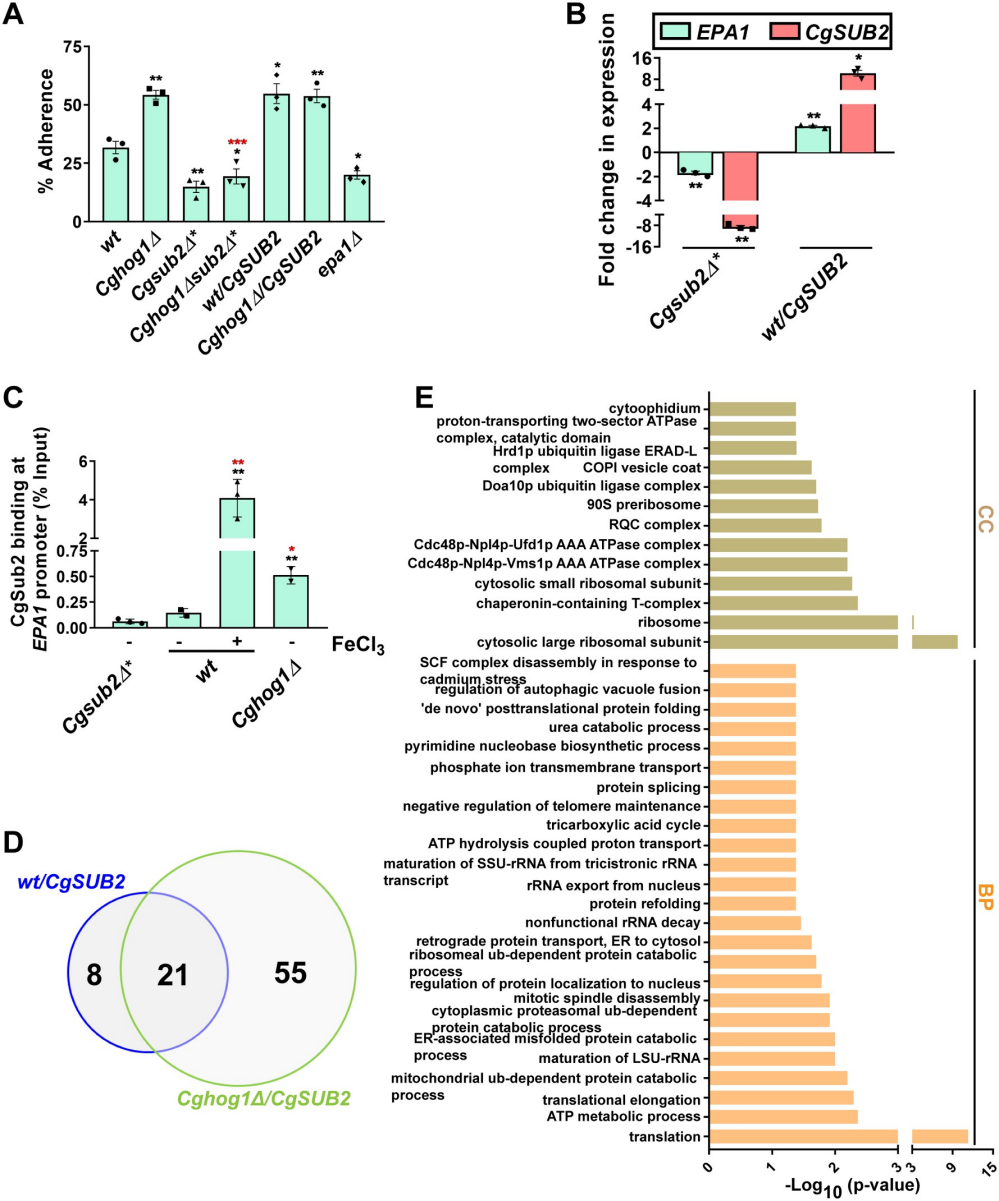
CgSub2 is required for *Cg* adherence to host epithelial cells. **A. Adherence analysis of indicated S**^**35**^**-labelled *Cg* cells to A-498 kidney epithelial cells.** For Cgsub2Δ* (YRK3294) mutants, the sub-culturing was done in YNB medium containing S^35^ mix, and cold methionine (2 mM) and cysteine (2 mM). Data represent mean ± SEM (n = 3). Black and red asterisks denote statistically significant differences in the percentage adherence between *wt* (YRK20) and indicated strains, and between *Cghog1Δ* (YRK964) and Cghog1Δsub2Δ* (YRK3372), respectively. *p ≤ 0.05; **p ≤ 0.01; ***, p ≤ 0.001, unpaired two-tailed Student’s *t*-test. **B. qRT-PCR-based measurement of *EPA1* and *CgSUB2* transcript levels.** Total RNA (500 ng), extracted from log-phase cultures of *wt* (YRK20), Cgsub2Δ* (YRK3294) and *wt/CgSUB2* (YRK2803) strains, using the acid phenol method, was used for cDNA synthesis, followed by real-time quantitative PCR amplification. Cgsub2Δ* cells were grown in methionine and cysteine-containing YNB medium. Transcript levels were quantified using the 2^-ΔΔ^C_t_ method. Data (mean ± SEM, n = 3) were normalized against *CgACT1* mRNA control, and represent fold change in *EPA1* and *CgSUB2* expression in indicated strains compared to *wt* (taken as 1.0). *p ≤ 0.05; **p ≤ 0.01, paired two-tailed Student’s *t*-test. **C. Anti-CgSub2 antibody-based chromatin immunoprecipitation (ChIP) analysis showing CgSub2 enrichment on *EPA1* promoter**. Log-phase *wt* (YRK20) and *Cghog1Δ* (YRK964) cells were grown in YNB or YNB medium containing 1 mM FeCl_3_ for 2 h, whereas Cgsub2Δ* (YRK3294; used as control) cells were grown in methionine and cysteine-containing YNB medium. Amplification values from ChIP samples were normalized with their respective input samples. Data represent mean ± SD (n = 2–3). Black and red asterisks denote statistically significant differences in CgSub2 binding at *EPA1* promoter in Cgsub2Δ* and indicated strains, and between *wt* and indicated strains, respectively. *, p ≤ 0.05; **, p < 0.01, unpaired two-tailed Student’s *t* test. **D. Venn diagram illustrating overlap in CgSub2 interactors between *wt/CgSUB2* (YRK2803) and *Cghog1Δ/CgSUB2* (YRK3013). E. Fungifun-based functional analysis of 55 CgSub2-interactors, that were uniquely present in *Cghog1Δ*.** Enriched gene ontology (GO) terms for Biological Process (BP) and Cellular Component (CC) are shown.

*Cg*, upon growth in the high-iron medium, activates *EPA1* [codes for the major adhesin contributing to host adherence in vitro; [37]] expression and displays elevated adherence to Lec2 ovary epithelial cells [4]. Further, *Cghog1Δ* contained increased *EPA1* levels, and exhibited higher adherence to Lec2cells [4]. Since elevated adhesin expression may confer an advantage in the iron-rich host gut by promoting host adhesion, and impeding clearance by the host [24,38], we next asked if CgSub2 regulates *EPA1* expression. For this, we performed three experiments. First, we showed that *CgSUB2* deletion and overexpression led to decreased and increased adherence, respectively, to A-498 kidney epithelial cells, compared to *wt* (Fig 4A). The *epa1Δ* mutant (used as control) showed 1.5-fold lower adherence than *wt* (Fig 4A). Importantly, the increased adherence of *Cghog1Δ* is predominantly mediated by Epa1, as *Cghog1Δepa1Δ* was hypo-adherent (S4D Fig). We also verified that the presence of methionine and cysteine in the medium had no effect on *Cg* adherence to A-498 cells, as *wt* grown in the medium lacking or containing methionine and cysteine exhibited similar adherence (S4E Fig). Further, Cghog1Δsub2Δ* and *CgSUB2* overexpressing-*Cghog1Δ* strains exhibited 2.5-fold less and similar adherence, respectively, to A-498 cells, compared to *Cghog1Δ* (Fig 4A), thereby linking increased adherence of *Cghog1Δ* to CgSub2. These results, along with diminished intracellular iron levels of *Cghog1Δ*, upon *CgSUB2* conditional deletion (Fig 3B), indicate that CgHog1 and CgSub2 interact genetically. Importantly, *CgSUB2* conditional deletion, *CgSUB2* overexpression and *CgHOG1* deletion also rendered *Cg* cells hypoadherent, hyperadherent and hyperadherent to human stomach epithelial (AGS) cells, respectively (S4F Fig), indicating that CgSub2 is a positive regulator of adhesion to both stomach and kidney epithelial cell types.

Second, to determine if adhesion changes co-relate with *EPA1* transcription, we performed qRT-PCR. The 2-fold lower and 2-fold higher *EPA1* transcript levels in Cgsub2Δ* (*CgSUB2*-conditional knockout) and *wt*/*CgSUB2*, (*CgSUB2*-overexpressing strain), respectively (Fig 4B), highlight the opposite effects of *CgSUB2* deletion and overexpression on *EPA1* expression, and attribute the *Cg* adherence potential predominantly to *EPA1* levels. Notably, *EPA1* expression has been reported to be lower, higher and higher in iron-limited, iron-surplus and *CgHOG1*-deleted conditions, respectively [4], with Epa1 being the major adhesin for *Cg* adherence in vitro [37].

Further, similar to *Cghog1Δ*, *CgSUB2* overexpression also led to increased caecum colonization at day 1-post infection in the gastrointestinal candidiasis model (S5 Fig). Contrarily, *CgSUB2* loss attenuated the *Cg* ability to colonize and survive in the stomach, ileum, caecum and colon, as reduced fungal CFUs were recovered from Cgsub2Δ*-infected mice, compared to *wt*-infected mice at both day 1 and day 4 post-infection (S5 Fig). Since *wt/CgSUB2* CFUs in all organs were also lower than *wt* CFUs at day 4 post-infection (S5 Fig), similar to *Cghog1Δ* mutant (Fig 1C), it is unlikely that *CgSUB2* overexpression confers any significant survival advantage to *Cg*, despite higher initial caecum colonization in the gastrointestinal candidiasis model. Besides implicating CgSub2 in organ colonization and *Cg* survival in the host gut, these results also underscore that the increased adherence in vitro does not necessarily translate into elevated colonization and increased survival in vivo. This could in part be due to *Cg* colonization of the gut involving at least two steps; adherence and persistence, with an ability to withstand stress and survive in the host tissue being a prerequisite for the latter step. It is possible that *Cghog1Δ* and *wt/CgSUB2*, due to diverse stress susceptibility [4] and ectopic *CgSUB2* overexpression, respectively, are attenuated for survival post-adherence, thereby showing impaired gut colonization, compared to the *wt* strain (Figs 1C, S1B and S5). In this context, it is worth noting that increased *EPA1* expression has recently been implicated in macrophage activation, elevated secretion of the pro-inflammatory cytokine IL-1β and poor *Cg* survival in human macrophages [39]. Therefore, it is possible that while higher *EPA1* expression is beneficial for the initial *Cg* adherence to host tissues, it is detrimental for long-term persistence and survival, as Epa1 cell surface exposure may activate the host defense system against *Cg*. Consistent with this, *Cg* clearance in the murine systemic candidiasis model is dependent upon the recognition of three adhesins including Epa1, by the natural cytotoxic receptor NCR1 on Natural Killer cells [40].

Third, we performed ChIP-qPCR to examine if CgSub2 directly regulates *EPA1* expression. A 3.5- and 28-fold increase in CgSub2 occupancy on *EPA1* promoter in *Cghog1Δ* and high-iron-grown *wt*, respectively, compared to regular-iron-grown *wt* (Fig 4C) indicated that CgSub2 enrichment on *EPA1* promoter may contribute to *EPA1* activation, upon both *CgHOG1* deletion and growth in the surplus-iron environment [4]. Notably, Sub2 in *S*. *cerevisiae* binds RNA and unwinds RNA:DNA duplexes [41]. Therefore, it is possible that CgSub2 recruitment on *EPA1* promoter may be mediated by another protein/complex. Nevertheless, these results altogether underscore that CgHog1 and CgSub2 modulate *EPA1* expression and host adhesion negatively and positively, respectively, and that, excess iron stimulates CgSub2 phosphorylation, CgSub2 enrichment on *EPA1* promoter and *EPA1* transcription.

### Identification of CgSub2 interactome

To unveil the molecular basis underlying CgHog1- and CgSub2-controlled *EPA1* expression, we profiled CgSub2 interactome in the presence and absence of CgHog1. We identified 29 and 76 CgSub2-interacting proteins in *wt* and *Cghog1Δ*, respectively, including 21 common interactors (Fig 4D and S3 Table). Notably, of 29 CgSub2-interactors identified in *wt*, 7 were earlier found as CgHog1 interactors (Fig 2B and S1 and S3 Tables), reinforcing CgHog1-CgSub2 interaction.

Functional analysis of identified proteins revealed CgSub2 interactors to primarily belong to ‘Translation’, ‘rRNA export from Nucleus’, and ‘Maturation of SSU-rRNA from tricistronic rRNA transcript (SSU-rRNA, 5.8S rRNA, LSU-rRNA) processes (Fig 4E and S4 Table), underscoring CgSub2 functions in RNA metabolism. CgSub2 interactome in *wt* was specifically enriched for GO-BP terms ‘Intracellular protein transport’ and Glycolytic process’ (S4 Table). Importantly, the unique enrichment of ‘Chaperonin-containing T-complex’, ‘COPI vesicle coat’ and ‘Cdc48p-Npl4p-Ufd1p AAA ATPase complex’ GO terms for Cellular Component (S4 Table), and the 2.6-fold larger interactome of CgSub2 in the absence of CgHog1 (Fig 4D and 4E) may reflect several new interactions of CgSub2, including those with constituents of the protein-folding machinery. Consistently, the ‘Protein refolding’ BP-term was uniquely enriched in CgSub2 interactome in *Cghog1Δ* (S4 Table). In this context, it is worth noting that Hog1 in *S*. *cerevisiae* promotes survival of the stress caused by accumulation of the unfolded/misfolded proteins [42,43]. It thus is possible that CgHog1 deletion may result in ER stress and activation of the unfolded protein response pathway in *Cg*, and that, CgSub2 may facilitate protein refolding via direct or indirect interaction with protein chaperones. Of note, three unique chaperone interactors of CgSub2 viz., Hsp60, Hsp70 and Hsp90, identified in the *Cghog1Δ* mutant background (S3 Table) are likely to aid this process. However, the validation of CgSub2-CgHsp protein interaction as well as misfolded protein accumulation in *Cghog1Δ* need to be demonstrated to support this hypothesis.

### CgHog1 negatively regulates CgSub2-CgHtz1 interaction

From identified interactors of CgSub2, we selected CgHtz1, a variant of the core histone H2A, for further analysis for four reasons: (i) CgHtz1 interacted with CgSub2 uniquely in *Cghog1Δ*; (ii) Htz1 in *S*. *cerevisiae* regulates subtelomeric and telomeric gene expression [44]; (iii) Htz1 occupancy facilitates optimal transcription activation for a subset of genes with inactive promoters [45]; and (iv) high environmental iron relieves the subtelomeric adhesin gene silencing in *Cg* [4]. Of note, CgHtz1 displayed 64% and 63% identity to the canonical histone H2A protein encoded by two 396 *nt*-long ORFs, *CAGL0K11440g* (*CgHTA1*), and *CAGL0C04411g* (*CgHTA2*), respectively, in *Cg* (http://www.candidagenome.org/;S6 Fig). CgHta1 and CgHta2 differ from each other in one amino acid at the 9^th^ position (S6 Fig).

**Fig 5:**
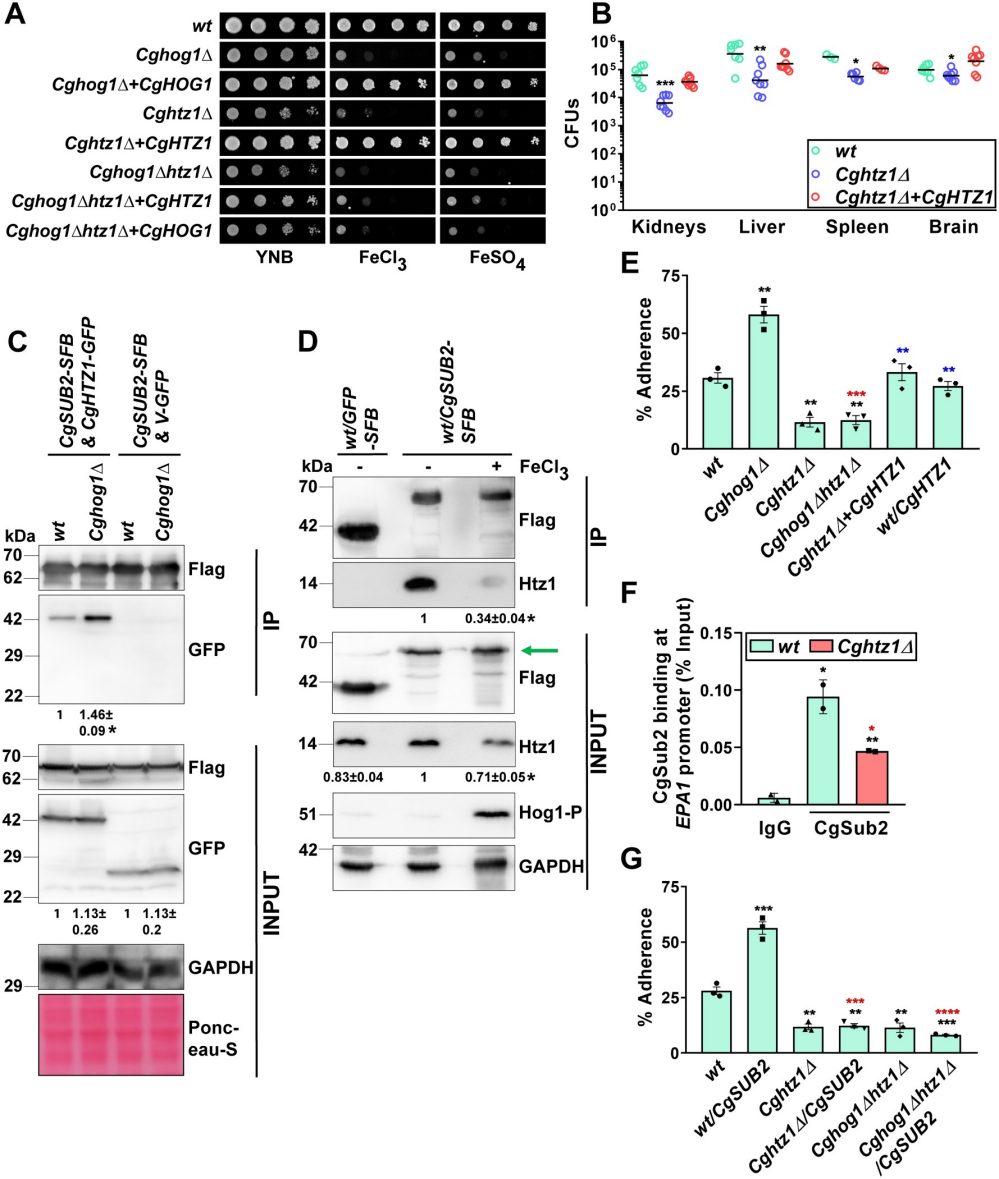
CgHog1 negatively regulates CgSub2-CgHtz1 interaction. **A. Serial dilution spotting assay showing sensitivity of indicated strains to ferric chloride (3 mM) and ferrous sulfate (20 mM). B. *Cghtz1***Δ **(YRK4392) survival analysis in systemic candidiasis model.** Overnight YPD medium-grown strains were injected intravenously into female BALB/c mice. At 7^th^ day post-infection, mice were sacrificed, indicated organs were collected and fungal burden was determined. Circles and bars denote colony-forming units recovered from an individual mouse, and the CFU geometric mean (n = 3–8), respectively, for each organ. *, p ≤ 0.05; **, p < 0.01; ***, p ≤ 0.001, Mann-Whitney U-test. **C. A representative Western blot showing CgSub2-CgHtz1 interaction**. Whole cell lysates (3 mg) of *wt* and *Cghog1*Δ strains expressing CgSub2-SFB, CgHtz1-GFP or Vector (V)-GFP were incubated with S-protein beads, followed by sample resolution on 15% SDS-PAGE and immunoblotting. 100 μg, 100 μg and 60 μg protein were loaded for CgSub2, CgHtz1 and CgGapdh detection, respectively, in input samples. Fold-increase in CgSub2-CgHtz1 interaction (mean ± SEM; n = 3) in *Cghog1*Δ, compared to *wt* (taken as 1.0), is shown underneath the blot. *, p ≤ 0.05, paired two-tailed Student’s *t* test. *wt/CgSUB2&CgHTZ1*, *Cghog1Δ/CgSUB2&CgHTZ1*, wt/*CgSUB2&V* and *Cghog1Δ/CgSUB2&V* strains correspond to YRK3872, YRK3979, YRK3870 and YRK3871 strains, respectively. IP, Immunoprecipitated samples. **D. A representative Western blot showing diminished CgSub2-CgHtz1 interaction under high-iron conditions.** *wt* cells expressing *CgSUB2-SFB* (YRK2803) were grown in YNB and ferric chloride (1 mM)-containing YNB medium for 2 h. Cells were lysed and whole cell lysates (3 mg) were incubated with streptavidin beads, followed by sample resolution on 15% SDS-PAGE and immunoblotting. *wt* cells expressing SFB-tagged GFP were used as negative control. Hog1-P indicates phosphorylated CgHog1 under iron-rich conditions (ferric chloride-containing medium). The arrow denotes CgSub2 protein band (65 kDa). Fold-decrease in CgSub2-CgHtz1 interaction (mean ± SEM; n = 3) in ferric chloride-grown *wt*, compared to YNB-grown *wt* cells (taken as 1.0), is shown underneath the blot. *, p ≤ 0.05, paired two-tailed Student’s *t* test. IP, Immunoprecipitated samples. **E. *Cg* adherence analysis to A-498 cells.** Data represent mean ± SEM (n = 3). Black, red and blue asterisks denote percentage adherence differences between *wt* and indicated strains, *Cghog1Δ* and *Cghog1Δhtz1Δ*, and *Cghtz1Δ* and *Cghtz1Δ-*complemented strain, respectively. **p ≤ 0.01; ***, p ≤ 0.001, unpaired two-tailed Student’s *t*-test. *wt*, *Cghog1Δ*, *Cghtz1Δ*, *Cghog1Δhtz1Δ*, *Cghtz1Δ+CgHTZ1* and *wt/CgHTZ1* strains, that correspond to YRK20, YRK964, YRK4392, YRK4390, YRK4737 and YRK4729, respectively, were grown in YNB medium. **F. ChIP analysis showing CgSub2 occupancy on *EPA1* promoter in *Cghtz1Δ* cells.** Log-phase *wt* (YRK20) and *Cghtz1Δ* (YRK4392) cells were grown in YNB medium. Amplification values from ChIP samples were normalized with their respective input samples. Data represent mean ± SD (n = 2). Black and red asterisks denote statistically significant differences in CgSub2 binding at *EPA1* promoter in indicated strains, compared to IgG control, and between *wt* and indicated strains, respectively. *, p ≤ 0.05; **, p < 0.01, unpaired two-tailed Student’s *t* test. **G. Adherence analysis of YNB-grown *CgSUB2*-overexpressing cells to A-498.** Data represent mean ± SEM (n = 3). Black and red asterisks denote percentage adherence differences between *wt* and indicated strains, and CgSub2-overexpressing *wt* and indicated strains, respectively. **p ≤ 0.01; ***, p ≤ 0.001; ****p ≤ 0.0001, unpaired two-tailed Student’s *t*-test. *wt*, *wt/CgSUB2*, *Cghtz1Δ*, *Cghtz1Δ/CgSUB2*, *Cghog1Δhtz1Δ* and *Cghog1Δhtz1Δ/CgSUB2* strains correspond to YRK20, YRK2803, YRK4392, YRK4909, YRK4390 and YRK4911 strains, respectively.

Since CgHtz1 functions were unknown, we first performed three experiments to examine its role in *Cg* pathobiology. First, we deleted *CgHTZ1*, and found that *Cghtz1Δ* grew slowly in minimum YNB medium, and displayed sensitivity to surplus-iron (Fig 5A). *Cghtz1Δ* also exhibited attenuated growth under DNA damage and oxidative stress conditions (S7A Fig). Second, we checked proliferation in human THP-1 macrophages via CFU-based assay, and found the *Cghtz1Δ* mutant to display 2-fold reduced replication, compared to *wt* cells (S7B Fig). Third, CgHtz1 was required for survival in mice, as 2- to 10-fold lower yeast colony-forming units were recovered from kidneys, liver, spleen and brain of *Cghtz1Δ-*infected mice, compared to *wt*-infected mice (Fig 5B). Importantly, *CgHTZ1* expression complemented all defects associated with *CgHTZ1* deletion (Figs 5A, 5B, S7A and S7B). Altogether, these data underscore CgHtz1 requirement for stress survival and pathogenesis of *Cg*.

Next, to examine CgHtz1 functions in CgHog1-mediated iron homeostasis, we first deleted *CgHTZ1* in *Cghog1Δ* background, and found that the double mutant *Cghog1Δhtz1Δ* was also growth-attenuated in the high-iron medium (Fig 5A). *Cghog1Δhtz1Δ* also exhibited increased susceptibility to DNA damage and oxidative stress (S7A Fig). Mutant complementation analysis revealed that *CgHTZ1* and *CgHOG1* expression in the *Cghog1Δhtz1Δ* mutant resulted in *Cghog1Δ* and *Cghtz1Δ* phenotypes, respectively (Figs 5A and S7A).

Next, we verified CgHtz1-CgSub2 interaction, and found higher interaction in *Cghog1Δ*, compared to *wt* (Fig 5C), thereby corroborating that CgHog1 negatively regulates CgSub2-CgHtz1 interaction. Notably, we found no interaction between CgHtz1 and CgHog1 (S7C Fig). Importantly, neither CgHtz1 levels were altered in *Cghog1Δ* (Fig 5C) nor CgHog1 phosphorylation was impaired in *Cghtz1Δ* (S7D Fig). However, high-iron led to diminished CgHtz1 levels (S7E Fig) and a decrease in CgSub2-CgHtz1 interaction (Figs 5D and S7F), thereby underscoring an inhibitory effect of surplus-iron on CgSub2-CgHtz1 interaction. Altogether, we draw three inferences from these data. First, CgHog1 and CgHtz1 do not interact physically. Second, CgHtz1 acts downstream of CgHog1 activation. Third, surplus-iron and CgHog1 have negative effect on CgSub2-CgHtz1 interaction, indicating that CgSub2 may activate *EPA1* transcription, via distinct mechanisms, upon *CgHOG1*-deleted and high-iron conditions.

### CgHtz1 is essential for *CgSUB2* overexpression-associated host adhesion

A strong and a weak CgSub2-CgHtz1 interaction under *CgHOG1*-deleted and high-iron conditions, respectively, prompted us to examine CgHtz1 functions in host adhesion. Unlike *CgSUB2*, *CgHTZ1* overexpression did not increase the *wt Cg* adherence (Fig 5E), despite containing 6-fold higher CgHtz1 levels, compared to endogenous CgHtz1 levels (S8A Fig). However, *Cghtz1Δ* displayed 2-fold lower adherence to A-498, compared to *wt*, while *CgHTZ1* deletion in *Cghog1Δ* background reduced the elevated adherence of *Cghog1Δ* to *Cghtz1Δ* levels (Fig 5E), suggesting that CgHog1-regulated host adherence is dependent on CgHtz1. The reduced adhesion of *Cghtz1Δ* was associated with diminished *EPA1* transcription (S8B Fig), and 2.5-fold reduced CgSub2 occupancy on *EPA1* promoter in the *Cghtz1Δ* mutant (Fig 5F). Further, *CgSUB2* overexpression, intriguingly, did not increase the adhesion of *Cghtz1Δ* and *Cghog1Δhtz1Δ* (Fig 5G), thereby highlighting CgHtz1 requirement for the elevated host adhesion associated with CgSub2 overexpression and *CgHOG1* deletion. Collectively, these results suggest that CgHtz1 is required for CgSub2 functions in host adhesion, and that, *CgHOG1*-deletion induced *EPA1* expression may be due to elevated occupancy of the CgSub2-CgHtz1 complex at the *EPA1* promoter.

### CgSub2 is essential for surplus-iron-induced host adherence

**Fig 6:**
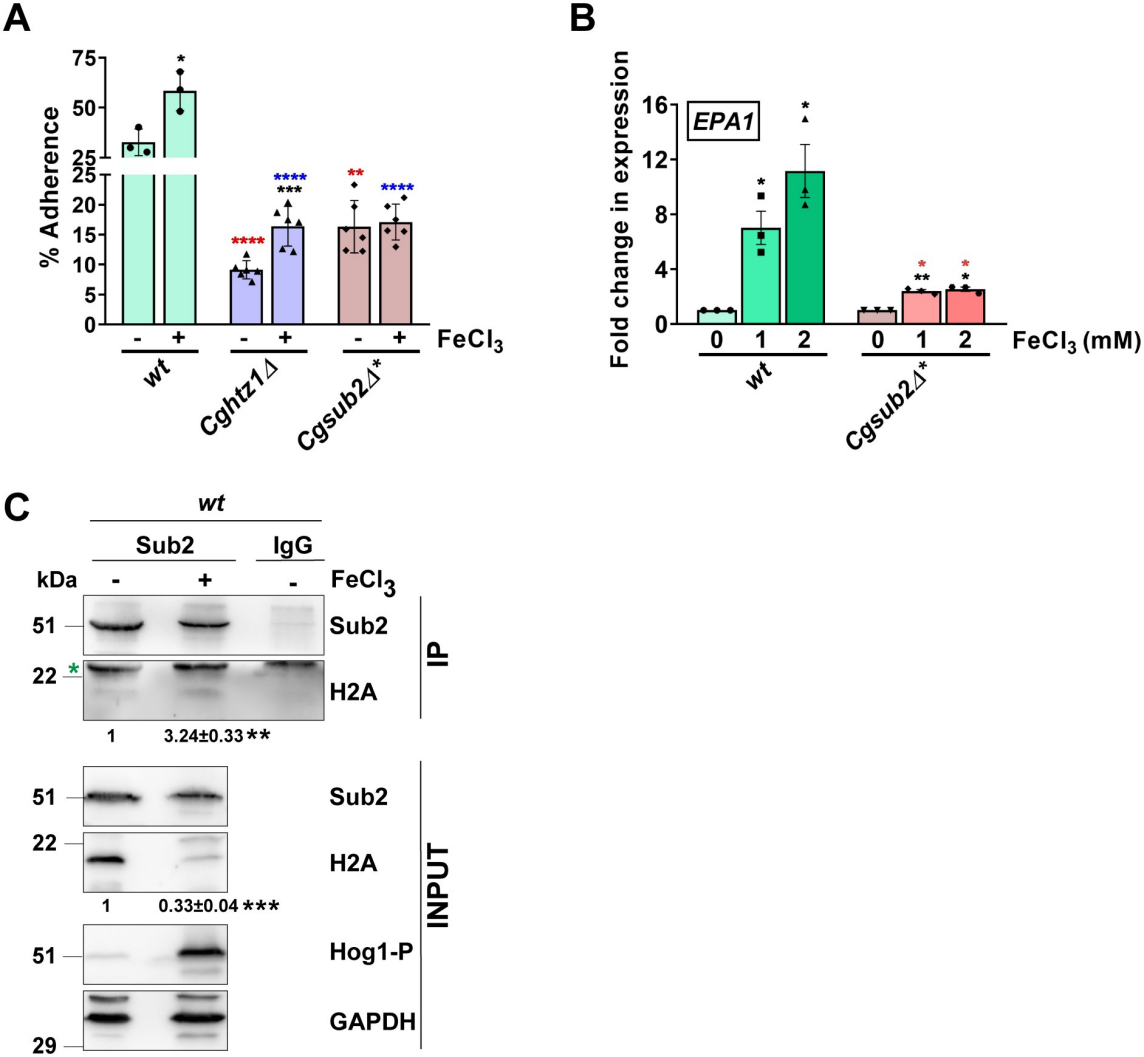
CgSub2 is required for surplus-iron-induced *Cg* adherence. **A. Adherence analysis.** *wt* (YRK20), *Cghtz1Δ* (YRK4392) and Cgsub2Δ* (YRK3294) cells were grown in YNB (regular-iron) and ferric chloride (500 μM)-containing YNB (high-iron) medium for 16 h, and labelled with S^35^. YNB medium used for subculturing Cgsub2Δ* also contained cold methionine (2 mM) and cysteine (2 mM). Data represent mean ± SEM (n = 3–6). Black asterisks denote percentage adherence differences between regular and high iron-grown *Cg* strains. Red and blue asterisks denote percentage adherence differences between regular iron-grown *wt* and indicated strains, and high iron-grown *wt* and indicated strains, respectively. *, p ≤ 0.05; **p ≤ 0.01; ***, p ≤ 0.001; ****p ≤ 0.0001, unpaired two-tailed Student’s *t*-test. **B. qRT-PCR-based *EPA1* expression analysis in indicated strains grown in YNB lacking or containing 1 mM and 2 mM ferric chloride.** Data (mean ± SEM, n = 3) were normalized against *CgACT1* mRNA control, and represent fold change in *EPA1* transcript levels in ferric chloride-grown cells, compared to respective YNB-medium grown cells (taken as 1.0). Black and red asterisks denote fold change in *EPA1* expression between YNB-grown *Cg* strains and indicated conditions, and similar-condition-grown *wt* (YRK20) and Cgsub2Δ* (YRK3294), respectively. *p ≤ 0.05; **p ≤ 0.01, two-tailed Student’s *t*-test. **C. A representative Western blot illustrating CgSub2-CgH2A interaction**. Lysates (15 mg) of *wt* cells, that were grown in YNB medium lacking or containing ferric chloride (1 mM) for 2 h, were incubated with anti-CgSub2 antibody, followed by sample resolution on 15% SDS-PAGE and immunoblotting. *wt* cell lysates incubated with 2.5 μg IgG-coated rProtein A-Sepharose beads were used as control. Fold-increase in CgSub2-CgH2A interaction (mean ± SEM; n = 4) in ferric chloride-grown *wt*, compared to YNB-grown *wt* cells (taken as 1.0), is shown underneath the blot. **, p < 0.01; ***, p ≤ 0.001, paired two-tailed Student’s *t* test. The green asterisk marks a non-specific band. IP, Immunoprecipitated samples.

Heat shock stress in *S*. *cerevisiae* disrupts histone-DNA interactions, and leads to nucleosome reassembly [46]. Therefore, in light of the high-iron-triggered CgSub2 enrichment on *EPA1* promoter, and decrease in CgSub2-CgHtz1 interaction, we hypothesized that *EPA1* transcriptional activation under high-iron conditions may arise from the canonical histone H2A replacing CgHtz1 as the CgSub2 interacting partner, with CgSub2 being indispensable for the high-iron-induced *EPA1* transcription. To test this, we performed three experiments. First, we measured adhesion of the surplus-iron-grown *wt*, Cgsub2Δ* (*CgSUB2* conditional knockout) and *Cghtz1Δ* strains to A-498 cells. Notably, like *Cghog1Δ* [4], Cgsub2Δ* did not respond to iron-excess by displaying elevated adhesion (Fig 6A), while growth in the high-iron medium led to an increase in the adherence of *Cghtz1Δ* (Fig 6A). Next, we checked *EPA1* expression, and found a substantial and a modest increase in *EPA1* transcript levels in high-iron-grown *wt* and Cgsub2Δ* cells, respectively, compared to respective regular iron-grown cells (Fig 6B). These results suggest that CgSub2 is pivotal to high-iron induced *EPA1* activation and elevated host adhesion.

Third, to check CgSub2 association with histone H2A under high-iron conditions, we performed co-immunoprecipitation assay. We found that CgSub2 interacted with H2A, with surplus-iron leading to a higher CgSub2-CgH2A interaction, despite a reduction in H2A levels (Fig 6C). Of note, decreased histone H2A levels under surplus-iron conditions may partly be due to general stress-responsive transcriptional downregulation of core histone genes in *Cg*, as the genotoxic stress is known to result in transcriptional downregulation of the histone H3 and H4 genes in *Cg* [47], and a reduction in histone mRNAs in *S*. *cerevisiae* [48]. Further, our co- immunoprecipitation results point towards the environment cue (iron abundance)-responsive interaction of CgSub2 with the canonical histone H2A or the histone H2A variant CgHtz1, which may potentially modulate chromatin dynamics. In this context, it is worth noting that Htz1-containing nucleosomes, due to their susceptibility to ejection, poise genes for rapid and full transcriptional activation, with heat shock-activated gene promoters displaying reduced Htz1 occupancy in *S*. *cerevisiae*, and oxidative stress causing Htz1 eviction from *cat-3* locus in *Neurospora crassa* [49,50]. Although the role of CgSub2-CgHtz1 and CgSub2-CgH2A interaction in *EPA1* regulation remains to be determined via CgHtz1 and CgH2A occupancy measurement on *EPA1* promoter under regular- and high-iron conditions, it is possible that the surplus extracellular iron in *Cg* leads to CgHtz1-CgH2A exchange at the *EPA1* promoter to activate *EPA1* transcription.

### Identification of phosphorylated amino acids in CgSub2

CgSub2 undergoes CgHog1-dependent phosphorylation (Fig 3E), exhibits decreased (Fig 5D) and increased (Fig 6C) interaction with CgHtz1 and CgH2A, respectively, under surplus-iron conditions, and is pivotal to high-iron induced increase in adherence to epithelial cells (Fig 6A). Therefore, we sought to identify all amino acid residues in CgSub2 that are phosphorylated. For this, we purified CgSub2-SFB from *wt*, *Cghog1Δ*, *Cgslt2Δ* and *Cghog1Δslt2Δ* strains, and mapped phosphorylation sites in CgSub2 by mass spectrometry analysis. The rationale for using *Cgslt2Δ* and *Cghog1Δslt2Δ* mutants was twofold. First, basal CgSub2 phosphorylation was observed in *Cghog1Δ* (Fig 3E). Second, CgSlt2, terminal MAPK of the cell wall integrity pathway, is required for iron homeostasis, and is activated in response to high iron [4]. Thus, a double-deletion strain *Cghog1Δslt2Δ*, that lacked both kinases, was used to investigate the cross talk between the iron-responsive CgHog1 and CgSlt2 MAPKs.

**Fig 7:**
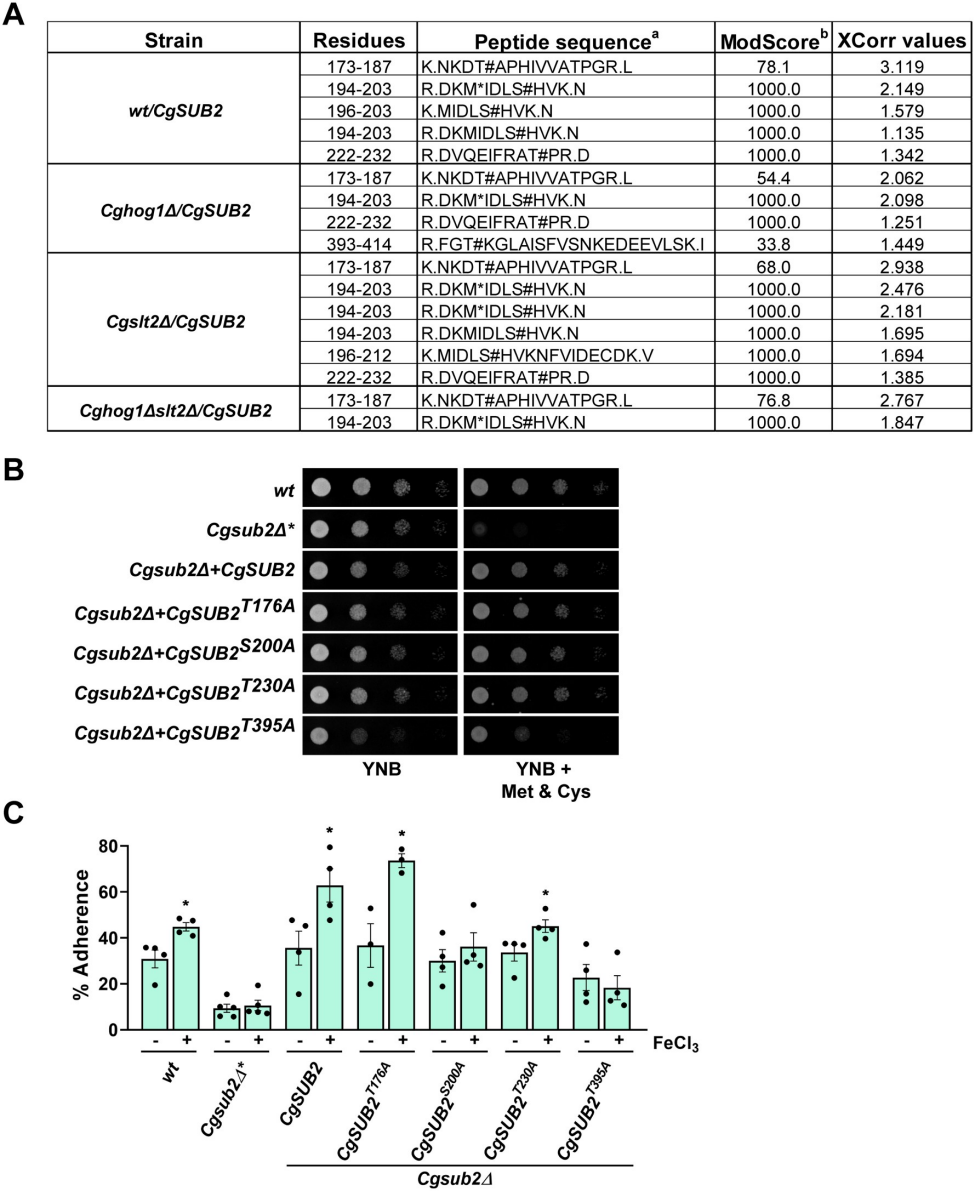
CgSub2 phosphorylation at Threonine-395 is important for CgSub2 functions. **A. Phosphosite-mapping of CgSub2 protein**. A list of identified phosphorylation sites in CgSub2 protein in indicated strains. ***a*,** the phosphorylated amino acid is marked with the hashtag symbol, #. ***b*,** ModScore represents confidence in the assigned phosphorylation sites. **B. Serial dilution spotting analysis showing growth of *CgSUB2* knockout cells, that were overexpressing CgSub2 or CgSub2 variants with indicated serine and threonine residues mutated to alanine.** *Cg* strains were grown overnight in CAA medium, collected, suspended in PBS and spotted on YNB and YNB medium containing methionine and cysteine. The Cgsub2Δ* strain (used as control) was grown in YNB medium containing 2 mM methionine and 2 mM cysteine. Plates were imaged after 2 days of growth at 30°C. *wt*, Cgsub2Δ*, *Cgsub2*Δ*+CgSUB2*, *Cgsub2*Δ*+CgSUB2*^*T176A*^, *Cgsub2*Δ*+CgSUB2*^*S200A*^, *Cgsub2*Δ*+CgSUB2*^*T230A*^ and *Cgsub2*Δ*+CgSUB2*^*T395A*^ strains correspond to YRK20, YRK3294, YRK6158, YRK6154, YRK6150, YRK6152 and YRK6156 strains, respectively. **C. Adherence analysis of *CgSUB2* knockout cells, that were overexpressing CgSub2 or CgSub2 variants, to A-498 cells via CFU-based assay.** For regular-iron condition, *Cg* strains were grown in YNB medium for 16 h, but for the Cgsub2Δ* strain (used as control) which was grown for 16 h in the YNB medium containing methionine (2 mM) and cysteine (2 mM). The high-iron medium contained 500 μM ferric chloride. *Cg* cells were collected and incubated with p-formaldehyde-fixed A-498 cells at 2:1 MoI. After 2 h incubation at room temperature, A-498 epithelial cells were washed with PBS and lysed in 1 ml PBS. The lysate was plated on YNB medium, and *Cg* colonies (Output) appeared were counted. To determine % adherence, the output CFUs were divided by input CFUs (Number of *Cg* cells infected to A-498 cells), and the number was multiplied by 100. Data represent mean ± SEM (n = 3–5). *p ≤ 0.05, unpaired two-tailed Student’s *t*-test. *wt*, Cgsub2Δ*, *Cgsub2Δ+CgSUB2*, *Cgsub2Δ+CgSUB2*^*T176A*^, *Cgsub2Δ+CgSUB2*^*S200A*^, *Cgsub2Δ+CgSUB2*^*T230A*^ and *Cgsub2Δ+CgSUB2*^*T395A*^ strains, correspond to YRK20, YRK3294, YRK6158, YRK6154, YRK6150, YRK6152 and YRK6156 strains, respectively.

We identified three, four, three and two phosphorylated residues in CgSub2 in *wt*, *Cghog1Δ*, *Cgslt2Δ* and *Cghog1Δslt2Δ*, respectively (Fig 7A). Threonine-176, Serine-200 and Threonine-230 were phosphorylated in three strains, *wt*, *Cghog1Δ* and *Cgslt2Δ* (Fig 7A). CgSub2 was also phosphorylated at an additional Threnonine-395 residue in *Cghog1Δ* (Fig 7A). Contrarily, Thr-230 phosphorylation was absent in *Cghog1Δslt2Δ* (Fig 7A), thereby highlighting CgHog1 and CgSlt2 indispensability for CgSub2 phosphorylation at Thr-230 residue. Notably, the NetPhos 3.1 tool predicted all four identified residues to be phosphorylated (https://services.healthtech.dtu.dk/services/NetPhos-3.1/). The identified residues, Thr-176, Ser-200 and Thr-230, and Thr-395, reside in the ATP-binding helicase domain and the Helicase conserved C-terminal domain of CgSub2, respectively (S9A Fig), with Thr-230 phosphorylation lacking in *Cghog1Δslt2Δ*, and Thr-395 phosphorylation being uniquely present in *Cghog1Δ* (Fig 7A). Importantly, while all four identified residues were conserved between *Cg* and *S*. *cerevisiae* Sub2, Thr-230 and Thr-395 were also conserved across their *Drosophila* and human counterparts [31] (S9B Fig).

To investigate the significance of CgSub2 phosphorylation in *Cg* biology, we mutated Thr-176, Ser-200, Thr-230 and Thr-395 to alanine, and checked if CgSub2 mutant proteins could perform the essential function of CgSub2 in *Cg* growth. For this, we replaced CgSub2 in the *Cgsub2Δ/CgSUB2* strain (expresses *CgSUB2* from *PDC1* promoter) with CgSub2^*T176A*^, CgSub2^*S200A*^, CgSub2^*T230A*^ and CgSub2^*T395A*^, and monitored cell growth in the YNB medium. We found that alanine replacements of three phosphorylated residues viz., Thr-176, Ser-200 and Thr-230, in CgSub2 had no effect on cell growth, as similar growth was observed for *Cgsub2Δ* carrying *CgSUB2*^*T176A*^, *CgSUB2*^*S200A*^, *CgSUB2*^*T230A*^ or *CgSUB2* (Fig 7B). Contrarily, compared to *Cgsub2Δ/CgSUB2*, *Cgsub2Δ/CgSUB2*^*T395A*^ grew slowly on YNB medium and YNB medium containing methionine and cysteine (Fig 7B), suggesting that Thr-395 in CgSub2 may be important for CgSub2 functions in cell growth. Expectedly, Cgsub2Δ* was unable to grow in methionine and cysteine-containing YNB medium (Fig 7B), reflecting CgSub2 essentiality. These data suggest that Thr-176, Ser-200 and Thr-230 phosphorylation in CgSub2 is dispensable for CgSub2 essentiality in *Cg*.

Next, we checked the effects of CgSub2 mutations on host adhesion. For this, we first examined if overexpression of CgSub2 mutant proteins results in hyperadherence in *wt* cells. We found that *wt*-expressing CgSub2^*T176A*^, CgSub2^*S200A*^, CgSub2^*T230A*^ and CgSub2^*T395A*^ displayed elevated adherence similar to *wt*-expressing CgSub2 (S9C Fig), suggesting that mutating these residues individually had no effect on host adhesion of *wt* cells that contain endogenous CgSub2. Secondly, we asked if CgSub2 phosphorylation at Thr-176, Ser-200, Thr-230 and Thr-395 residues is important to rescue the diminished adherence associated with *CgSUB2* conditional deletion. For this, we checked the adhesion of *Cgsub2Δ* expressing CgSub2^*T176A*^, CgSub2^*S200A*^, CgSub2^*T230A*^, CgSub2^*T395A*^ or CgSub2, to A-498 cells. We found that while all CgSub2 mutant proteins could complement diminished adherence of the *CgSUB2*-conditional knockout, the elevated adherence, upon growth in surplus-iron condition, was observed only upon expression of CgSub2^*T176A*^, CgSub2^*S230A*^ and CgSub2 proteins (Fig 7C). The similar adherence of *Cgsub2Δ/CgSUB2*^*S200A*^ and *Cgsub2Δ/CgSUB2*^*T395A*^ under both regular- and high-iron conditions (Fig 7C) implicate Ser-200 and Thr-395 amino acids in iron abundance-responsive CgSub2 phosphorylation and *EPA1* activation. Notably, Thr-395, that lies in the C-terminal helicase domain of CgSub2, was phosphorylated uniquely in *Cghog1Δ* (S9A Fig), with Thr-395 mutation to alanine impairing CgSub2 functions in cell growth (Fig 7A). Besides highlighting the importance of Thr-395 residue in proper functioning of CgSub2, these data point towards two distinct, basal and iron-induced phosphorylation-dependent functions, of CgSub2 in host adherence. Further studies are warranted to identify phosphorylated residues in CgSub2 under surplus-iron conditions, and determine identified residues’ phosphorylation significance for the helicase activity, *EPA1* transcriptional regulatory function and CgHtz1/CgH2A interaction of CgSub2. However, detection of the phosphorylated amino acids in CgSub2 in the *Cghog1Δ* mutant suggests that CgHog1 is unlikely to be the sole kinase that phosphorylates CgSub2. In this regard, it is worth noting that since our in vitro CgSub2 phosphorylation assays were carried out with whole-cell lysates of *wt* and *Cghog1Δ* strains (Fig 3E), it is possible that CgSub2 is phosphorylated by other CgHog1-interacting/activating kinases.

In summary, our findings report a nexus among a MAPK, a putative RNA helicase and a histone variant, that links adhesin gene expression with iron availability in *Cg*.

## Discussion

CgHog1 is the terminal kinase of the HOG phosphorelay system, and exposure to acid and osmotic stressors viz., sodium chloride, glycerol, sorbic acid and lactic acid, leads to CgHog1 phosphorylation and increased accumulation in the nucleus [15,51]. However, CgHog1 targets remain unidentified. Herein, we identify and functionally characterize an unanticipated CgHog1 substrate, a putative RNA helicase CgSub2. We show that CgSub2 is essential for high iron-invoked *EPA1* activation, with CgHog1 governing CgSub2 functions in host adhesion by negatively regulating CgSub2-CgHtz1 interaction.

*Cg* resides in the oxygen-poor, iron-rich gut, and *Cg* infections can be endogenous, with the gastrointestinal tract as a probable source [18,26,52–54]. Importantly, gut is also a major reservoir for resistance development against the mainstay antifungal drug, caspofungin [19]. In spite of this, factors facilitating *Cg* survival in the gut are largely unknown. *Cg* is known to colonize caecum [55], and the lactate dehydrogenase CgCyb2 is required for survival in the caecum [52]. *Cg* adhered better to Caco-2 intestinal cells under hypoxia due to increased expression of the Epa6 adhesin, with Epa6 also being pivotal to mouse gut colonization [56]. Besides implicating CgHog1 in *Cg* survival in the host gut, our study provides the first line of evidence on how iron abundance impacts *Cg* colonization and intestinal survival. *Cg* colonization was higher in the mouse caecum at day 1 post-infection in mice under surplus-iron conditions (Fig 1C). Notably, it has previously been shown that the high-iron diet-fed rat contain 3-fold increased luminal iron levels in the caecum and colon, compared to the regular-iron diet-fed rat, with the caecum luminal iron content being 4-fold higher than the colon luminal iron under both regular and high-iron diet conditions [57]. Further, consistent with the better *Cg* colonization of the caecum under high-iron conditions, the caecal load for *Cghog1Δ*, that displays higher adhesin expression and elevated adherence in vitro, was higher, compared to *wt*, in regular-iron diet-fed mice after 1 day of infection (Fig 1C), indicating that *Cghog1Δ* probably initially adheres better than *wt*. However, this increased colonization is inadequate to confer a long-term survival advantage, as CgHog1 is required for *Cg* survival in the gut. CgHog1 centrality for *Cg* survival in the host could partly be attributed to its role in governing many transcriptional regulatory networks, maintaining iron homeostasis, restraining adhesin expression and suppressing host cytokine production [4,51].

Human host infection is a complex multi-step process, wherein host adherence precedes pathogen internalization. Adherence to host tissues, which is mediated by multigene family of adhesins in *Cg*, is considered as a first step for fungal infection establishment [58]. *Cg* genome codes for 81 adhesins including several subtelomerically-encoded adhesins [59]. The subtelomeric adhesin genes including *EPA1* are subjected to SIR complex-mediated transcriptional silencing [58]. CgHog1 governs subtelomeric gene silencing, as *CgHOG1* deletion resulted in elevated expression of 26 adhesin genes including 18 subtelomeric genes [4]. It is possible that elevated *EPA1* expression, upon *CgHOG1* loss, is due to relief of subtelomeric gene silencing as well as altered CgSub2-dependent *EPA1* regulation. Of note, Hog1 activation is known to positively regulate telomeric gene silencing in *S*. *cerevisiae* [60]. Importantly, high environmental iron, that probably mimics the gut environment, also activates subtelomeric adhesin genes in *Cg* [4]. Our findings link these two signals mechanistically, and raise the possibility of CgHog1 aiding gut colonization by phosphorylating CgSub2, and triggering adhesin gene expression. In this context, it is worth noting that expression of the two other adhesin genes, *EPA3* and *EPA6*, was downregulated upon *CgSUB2* deletion, while *EPA6* transcript levels were higher in *CgSUB2-*overexpressing strain (*wt/CgSUB2*), compared to *wt* cells (S9D Fig). These results suggest that CgSub2 may contribute to *Cg* survival in the host gut by regulating expression of multiple adhesin genes.

Iron uptake is a limiting factor for *Cg* infections [34,61–63]. Owing to binding to high-affinity proteins including the transport protein transferrin and the storage protein ferritin, iron availability is highly restricted in the host [22]. The enriched-iron diet-fed mice and rat are known to have elevated iron levels in the liver and the spleen [64,65]. Our analysis of *Cg* survival in the systemic candidiasis model revealed no significant change in *Cg* CFUs in liver, spleen, kidneys and brains of regular- and high-iron diet-fed mice (Fig 1B), thereby raising two possibilities. First, there is ample iron availability for *Cg* in murine organs. Second, surplus iron does not promote *Cg* survival in the systemic candidiasis model. Further studies are warranted to test these possibilities.

*Cg* acquires iron via the high-affinity iron uptake system from iron salts and ferritin [34,63,66]. *CgSUB2* overexpression led to high intracellular iron levels, and increased expression of the subtelomerically-located *EPA1* gene (Figs 3B and 4B). In *S*. *cerevisiae*, the DEAD-box RNA helicase Sub2 is required for transcription elongation, genome stability, and export of mRNAs out of the nucleus [31,67]. Since iron metabolism is regulated post-transcriptionally, with the mRNA-degrading protein CgCth2 restricting iron consumption during iron-limited conditions, and its *S*. *cerevisiae* ortholog governing nuclear export and decay of mRNA transcripts of iron-utilization genes [62,63,68], it is possible that CgSub2 may control the export of iron homeostasis gene-encoding mRNAs. Further, Sub2 in *S*. *cerevisiae* is recruited to active chromatin by the pentameric THO complex in a RNA-dependent manner [69,70], whether a similar mechanism operates in *Cg* is worth examining.

Chromatin remodelling complexes (CRCs) modify chromatin functions by substituting canonical histones with histone variants [71]. Htz1 is a conserved variant of the canonical histone H2A, and involved in impeding the spread of heterochromatin [44]. We found that CgSub2 interaction with CgHtz1 and H2A was decreased and increased, respectively, under high-iron conditions, indicating histone exchange. Although CgHtz1 displacement at the chromatin (*EPA1* promoter) in high-iron-grown cells is yet to be demonstrated, our preliminary analysis suggests that CgSwr1 and CgINO80 CRCs may carry out this chromatin remodeling, as both *Cgswr1Δ* and *Cgino80Δ* (lack ATPase subunits of their respective CRCs), grew poorly in high-iron medium (S9E Fig), suggesting a defect in sensing and/or responding to surplus-extracellular iron. Notably, Swr1 and INO80 in *S*. *cerevisiae* are required for Htz1 deposition at euchromatin and Htz1 eviction from promoters during transcriptional activation, respectively [72–75]. Finally, our findings raise the possibility of the environmental cue-responsive *EPA1* locus-specific nucleosome composition, which could be triggered by post-translational modifications and/or reduction in cellular CgHtz1 levels, with H2A.Z (mammalian ortholog of CgHtz1) monoubiquitylation being associated with gene silencing [76].

**Fig 8:**
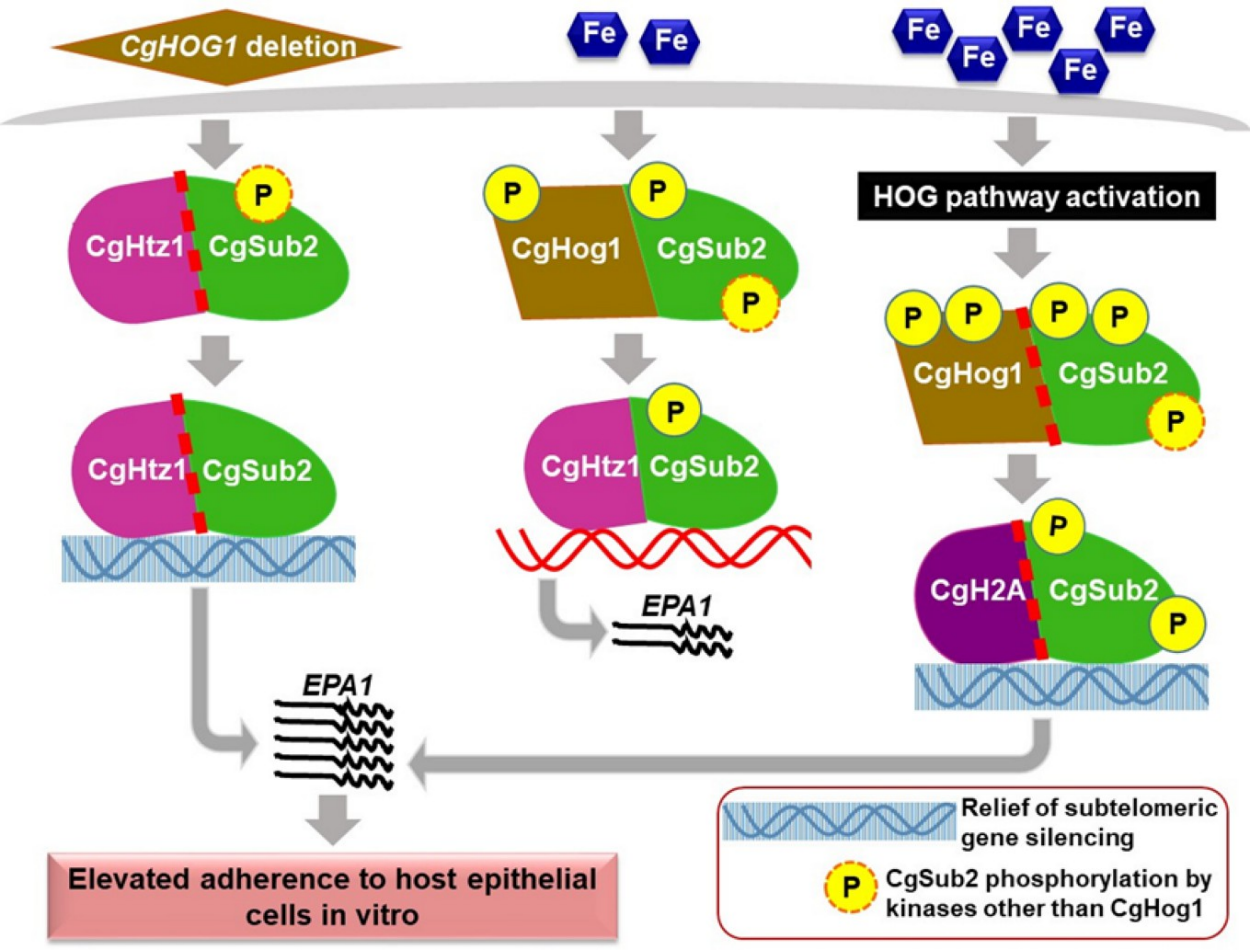
A schematic summarizing key findings of the study. *EPA1* transcription is restrained and activated under regular- and high-iron conditions, respectively, via subtelomeric gene silencing and CgHog1-CgSub2-CgHtz1/CgH2A axis-mediated regulation, with CgHog1 acting as a negative regulator of *EPA1* expression and host adherence. The dashed red line denotes elevated interaction between interacting proteins.

Altogether, based on our data, we propose that CgHog1 restrains *EPA1* expression by regulating subtelomeric adhesin gene silencing positively [4], and CgSub2-CgHtz1 interaction negatively. The latter probably adversely impacts CgSub2 recruitment on *EPA1* promoter, thereby restricting *EPA1* expression (Fig 8). Surplus-iron-induces CgSub2 phosphorylation, replaces CgHtz1 with CgH2A as CgSub2-interacting partner, as well as relieves subtelomeric gene silencing which triggers *EPA1* transcriptional activation, and increased *Cg* adherence to host epithelial cells in vitro (Fig 8). Finally, elevated *EPA1* levels may promote *Cg* colonization of the caecum during the initial stage of infection via increased epithelial cell adherence, without bestowing any long-term survival advantage to *Cg* in the host gut. Notably, increased *EPA1* expression has recently been shown to activate macrophages, leading to increased IL-1β secretion and diminished *Cg* survival in macrophages [39]. Additionally, *Cg* clearance in the systemic model was found to be dependent upon the recognition of three adhesins including Epa1, by the natural cytotoxic receptor NCR1 on Natural Killer cells [40]. Therefore, higher *EPA1* expression may be beneficial for the initial *Cg* adherence to host tissues, but may not aid long-term *Cg* survival in the host.

In conclusion, besides identifying functional links among CgHog1, CgSub2 and CgHtz1, our work underscores the CgHog1 signalling network dynamics, and have implications for *Cg* commensalism in the gastrointestinal tract and its pathogenesis, with iron availability in individual organs probably contributing to commensal and pathogenic life styles.

## Materials and methods

### Ethics statement

Mice infection experiments were performed at the Experimental Animal Facility of Centre for DNA Fingerprinting and Diagnostics (CDFD), Hyderabad, India in accordance with guidelines of the Committee for the Purpose of Control and Supervision of Experiments on Animals, Government of India. All procedures were designed to minimize mice suffering, and were approved by the Institutional Animal Ethics Committee [EAF/RK/21/2023 (for gastrointestinal candidiasis model) and EAF/RK/23/2023 (for systemic candidiasis model)].

### *Cg* growth and viability analysis

The vaginal isolate BG2 was used as the *Cg* wild-type (*wt*) strain, and all mutants were derived from this *wt* strain. *Cg* strains were routinely cultured in rich YPD medium (1% Yeast extract, 2% Peptone and 2% Dextrose) at 30°C. The effect of iron on cell growth was assessed in synthetic YNB or CAA medium. YNB medium consisted of 0.67% YNB (Yeast Nitrogen Base; with ammonium sulfate and without amino acids, and contains 1.23 μM ferric chloride) and 2% dextrose [4]. To prepare the CAA medium, 0.6% casamino acids was added to the YNB medium. To achieve logarithmic-phase *Cg* cells, overnight-grown *Cg* strains were grown for 3–5 h. Strains used are listed in the S5 Table.

For stress susceptibility analysis, serial dilution spotting assay was performed. Briefly, overnight-grown *Cg* cells were collected, washed, suspended in PBS and OD_600_ was normalized to 1.0. Next, cultures were serially diluted 10-fold in PBS and 3 μl of each dilution was spotted on medium lacking or containing different compounds. After 1–3 days of incubation at 30°C, growth was recorded using the Gel Doc Imaging System.

For time-course analysis, overnight YNB-grown cultures were re-inoculated in fresh YNB lacking or containing methionine (2 mM) and cysteine (2 mM) medium at an initial OD_600_ of 0.2. Culture absorbance was measured at 600 nm at regular intervals till 48 h, and OD_600_ values were plotted against time.

### Gene cloning and disruption

*Cg* ORFs were replaced at the genomic locus with nourseothricin resistance-conferring *nat1* gene (encodes nourseothricin acetyltransferase enzyme) using homologous recombination, as described previously [4]. *Cg* deletion strains were confirmed by PCR. *Cghog1Δ* mutant was used to generate *Cghog1Δslt2Δ*, *Cghog1Δhtz1Δ*, Cghog1Δsub2Δ* (Conditional deletion of *CgSUB2* in *Cghog1Δ* mutant background) and *Cghog1Δepa1Δ* double-deletion strains.

To generate *CgSUB2-*conditional knockout, *CgSUB2* ORF (*CAGL0L06908g*; 1.32 kb)- was tagged with the SFB (S protein-Flag-Streptavidin-binding peptide)-epitope-encoding sequence at the C-terminus via cloning in XbaI-XmaI sites in the pRK1349 plasmid. Next, CgSub2-SFB was sub-cloned in XbaI-SalI sites in the pCU-MET3 plasmid (pRK1011) under the methionine-repressible *CgMET3pro* promotor and transformed into *wt* cells. Transformants were selected for uracil prototrophy and colony purified. *CgSUB2* at the genomic locus of one purified transformant was replaced with the *nat1* gene using homologous recombination-based strategy. To achieve this replacement, 5’ and 3’ UTRs (about 650 bp) of the *CgSUB2* gene were PCR amplified using *wt* genomic DNA as a template. Next, both 5’ and 3’ gene UTRs were fused to one half each of the *nat1* gene, which were PCR amplified from the *nat1* gene-containing plasmid pRK625. The two *nat1* amplified fragments shared about 300–350 bp of complementary region. Fused PCR products were co-transformed into the uracil prototrophic *wt* transformant expressing *CgSUB2* ectopically from *CgMET3Pro*. Transformants were plated on YNB medium and incubated at 30°C for 12–16 h, followed by replica plating on nourseothricin (200 μg/ml)-containing YNB medium, containing monosodium glutamate as nitrogen source, in place of ammonium sulphate. After 24 h incubation at 30°C, nourseothricin-resistant colonies were purified and checked for *CgSUB2* replacement at the genomic locus with *nat1* by PCR. For growth analysis, *CgSUB2-SFB* expression was repressed by growing *CgSUB2-*conditional knockout in the presence of 2 mM methionine and 2 mM cysteine. *CgSUB2-SFB*, cloned under *CgPDC1* promoter in the pRK1349 plasmid, was used for overexpression and protein interaction analyses.

For complementation studies, *CgHTZ1* (*CAGL0E02315g*; 0.40 kb) was cloned in SpeI-XmaI sites in the pRK1349 plasmid. For *CgHOG1* complementation, *CgHOG1* (*CAGL0M11748g*; 1.34 kb) cloned in BamHI-XmaI sites in the pRK74 plasmid, was used, as described previously [4]. *CgHTZ1* were tagged with GFP-encoding sequence at the C-terminus via cloning in XbaI-SpeI sites in the pRK1965 plasmid. *CgHOG1* was cloned in BamHI-XmaI sites in the pRK1131 plasmid for N-terminal SFB tagging. Specific residues in *CgSUB2* or *CgHOG1* were mutated using mutagenic primers. All clones were verified by PCR, restriction digestion, sequencing and complementation analysis. The plasmids and primers used are listed in S6 and S7 Tables, respectively.

### Protein extraction and immunoblotting

Log-phase *Cg* cells were grown in YNB or YNB medium containing 2 mM FeCl_3_ for 2 h, collected, washed with chilled PBS and were snap-frozen. Frozen cells were lysed with 0.5 mm glass beads in protein extraction buffer [Tris-HCl (50 mM; pH 7.5), EDTA (2 mM), sodium fluoride (10 mM), sodium orthovanadate (1 mM) and 1 X protease inhibitor cocktail]. Cell lysates were collected after centrifugation at 13,000 rpm for 10 min at 4°C, and protein concentration was estimated using BCA kit. For protein analysis, 120, 100, 100, 100, 80 and 60 μg protein were loaded to detect phosphorylated CgHog1, total CgHog1, CgSub2, CgHtz1, CgSub2-SFB and CgGapdh, respectively.

For immunoprecipitation, *Cg* cell lysates were prepared in lysis buffer [Na_2_HPO_4_ (15 mM), NaCl (150 mM), Triton X- 100 (2%), SDS (0.1%), sodium deoxylcholate (0.5%), EDTA (10 mM), NaN_3_ (0.02%)] using glass beads. rProtein A-Sepharose Fast Flow beads (GE Healthcare) or rProtein G-Sepharose Fast Flow beads were coated with appropriate antibodies for 6 h in lysis buffer at 4°C, followed by addition of pre-cleared cell lysates (5 to 15 mg). After 12 h incubation at 4°C, beads were washed 5 times with lysis buffer to remove non-specific interactions, and bead-bound proteins were resolved on a SDS-PAGE gel after boiling in SDS-PAGE sample buffer at 95°C for 10 min. Gels were transferred to PVDF membrane and probed with appropriate antibodies. CgHog1 and phosphorylated CgHog1 were detected using anti-CgHog1 (raised in mice) and phospho-p38 MAPK (Thr180/Tyr182) antibody (Cell Signaling Technology #4511L) antibodies, respectively. All antibodies used are listed in the S8 Table.

### Mice maintenance and infections

The BALB/c and C57BL/6 mice are routinely maintained on iron-adequate diet containing 192 mg iron/kg for systemic and gastrointestinal candidiasis, respectively. For iron abundance experiments, the mice were fed the formulated identical diet but for the iron content. The pellet diet contained two iron concentrations, regular-iron (190 mg/kg) or excess-iron (500 mg/kg). Mice were fed experimental diets for 10 days, prior to *Cg* infection, and were maintained on the same diet during infection studies. Mice had free access to water and food during the entire study. No difference in weight or diet consumption was observed for the experimental mice. Mice were euthanized by CO_2_ inhalation before organ collection.

For the systemic candidiasis model, overnight YPD-grown *Cg* (4 X 10^7^ cells; 100 μl cell suspension in PBS) were injected into the tail vein of 6–8 week-old female BALB/c mice. At 1 and 4^th^ day post-infection (dpi), mice were sacrificed and four organs, kidneys, liver, spleen and brain, were collected. Organs were homogenized in PBS and plated on penicillin and streptomycin-containing YPD medium. *Cg* colonies were counted after 2 days of growth at 30°C.

For gastrointestinal candidiasis model, 6–8 week-old female C57BL/6 mice were given three antibiotics, tetracycline (1 mg/ml), streptomycin (2 mg/ml) and gentamicin (0.1 mg/ml), in drinking water for 7 days, prior to infection. Antibiotic-treated C57BL/6 mice were orally inoculated by a single gavage with overnight YPD-grown *Cg* (200 μl PBS cell suspension; 2.5 X 10^8^ cells) using a 24-gauge feeding needle. At 1 or 4 dpi, mice were sacrificed, dissected to separate individual gastrointestinal organs, and four organs, stomach, ileum, caecum and colon were collected. Organs were homogenized in NETN tissue lysis buffer [Tris HCl (20 mM; pH 8.0), NaCl (100 mM), EDTA (1 mM) and Nonidet P-40 (0.5%)] by three rounds of gentle mechanical shearing on Fastprep-24 (MP biochemicals). Fungal bioburden in organ lysates was calculated by plating appropriate dilutions of organ homogenates on YPD medium containing penicillin and streptomycin. *Cg* colonies were counted after 2 days of growth at 30°C.

### Mass spectrometry (MS) analysis

For CgHog1 interactome analysis, the *Cghog1Δ* mutant expressing either the N-terminally SFB (S protein-Flag-Streptavidin-binding peptide)-tagged CgHog1 (*Cghog1Δ/CgSFB-HOG1*) or the SFB tag (*Cghog1Δ/V*) were grown in CAA medium (contains 1.23 μM ferric chloride; regular- iron medium) for 12 h. Next, the *Cghog1Δ/CgSFB-HOG1* culture was starved for iron for 12 h via growth in 50 μM BPS (bathophenanthroline disulfonate; an extracellular iron chelator)-containing CAA medium. After washes, the iron-starved culture was further incubated in the CAA (regular-iron) medium and CAA medium supplemented with either 50 μM BPS (low-iron), or sodium ascorbate (1 mM) plus ferrous ammonium sulfate (500 μM) (high-iron) media for 2 h at 30°C. The *Cghog1Δ/V* strain, grown in CAA medium, was used as a control. After 2 h growth, cells were washed and lysed in NETN buffer [Tris-HCl (20 mM; pH 8.0), NaCl (100 mM), EDTA (1 mM) and Nonidet P-40 (0.5%)] using glass beads. Whole cell lysates were subjected to tandem affinity purification with streptavadin-agarose and S-protein-agarose beads. Briefly, 8 mg protein per lysate sample was first incubated with streptavidin beads for 2 h at 4°C, followed by elution in the biotin (2 mg/ml)-containing buffer. The resulting supernatant was incubated with S-protein agarose beads for 2 h at 4°C. After washes, beads were boiled, and proteins were resolved on a 10% SDS-PAGE gel till bromophenol blue dye in the sample buffer entered about 3 mm into the gel. The gel was stained with Coomassie Brilliant Blue and destained. A portion of the gel displaying protein bands was excised, placed in water and sent to the Taplin Biological Mass Spectrometry (MS) facility at Harvard Medical School, Boston, USA for protein identification via LC-MS/MS analysis. Samples were prepared and sent from two independent biological replicates.

For CgSub2 interactome analysis, overnight cultures of *wt* or *Cghog1Δ* expressing C-terminally SFB tagged CgSub2 (CgSub2-SFB) were grown in CAA medium for 5 h at 30°C in CAA medium. *wt*-expressing GFP (GFP-SFB) was used as control. Cells were lysed using glass beads. CgSub2 was purified from cell lysates (8 mg), using streptavadin-agarose and S-protein-agarose beads, as described above for CgHog1 interactor analysis, and samples were sent to the Taplin Biological Mass Spectrometry (MS) facility for LC-MS/MS analysis.

For MS data analysis, two criteria were applied. First, proteins, that were found in control lysate samples, were not considered, as these represented proteins bound non-specifically to the affinity matrices. Second, proteins that were represented by a minimum of two total peptides in each replicate sample, were selected.

For phosphorylation site identification in CgSub2, CgSub2-SFB was pulled down from whole cell lysates (15 mg) of CAA medium-grown *wt*, *Cghog1Δ*, *Cgslt2Δ* and *Cghog1Δslt2Δ* strains expressing *CgSUB2-SFB* using the two-step pull down assay involving lysate binding with streptavidin beads, followed by elution in biotin solution, and incubation of the first-step-eluate with S-protein agarose beads. Pulled-down protein samples were resolved on 12% SDS-PAGE. The 65 kDa band corresponding to CgSub2-SFB was cut from the gel, and sent to Taplin MS facility, Harvard Medical School, Boston for identification of phosphorylated residues in CgSub2 protein.

### Intracellular iron estimation

The intracellular iron content was measured by flame atomic absorption spectrometry (AAS). YNB-grown log phase *Cg* (50 OD_600_) were collected and washed twice with HPLC-grade water. Cells were lysed overnight in 30% HNO_3_ at 95°C, and supernatants were collected after centrifugation at 13000 rpm for 10 min. Iron content in supernatant samples was determined, via AAS, using the standard curve generated for different iron concentrations in 5% HNO_3_.

### CgSub2 and CgHog1 purification

*CgHOG1* was cloned in the pET28a^+^ plasmid between BamHI and SacI Sites, and transformed into the *E*. *coli* BL21 (DE3) strain. A purified transformant was induced with 1 mM IPTG for *CgHOG1* expression at 37°C for 3 h. Cells were collected, suspended in lysis buffer [Tris-HCl (50 mM; pH 7.5), NaCl (500 mM), β-ME (5 mM), Triton X-100 (1%) and Glycerol (10%)] and sonicated using Diagenode bioruptor plus. After sample centrifugation at 12,000 rpm for 5 min, the pellet was suspended in 6 M urea and resolved on 12% SDS-PAGE. The recombinant CgHog1 protein band was cut from the gel, gel slices were crushed in the gel elution buffer [Tris-HCl (25 mM; pH 8.8), glycerol (5%), SDS (1%) and glycine (0.24 mM)] using motor and pestle, and were incubated overnight at 37°C. Samples were centrifuged at 13,000 rpm for 15 min, pellet was suspended in gel elution buffer and the extraction process was repeated 3 times. Next, five volumes of chilled acetone were added to eluates and samples were spun down after overnight storage stored at -20°C. The pellet was washed with acetone, dried and solubilized with 10 mM Tris-HCl (pH 8.8) and 0.1% SDS. The soluble protein was collected after centrifugation at 12,000 rpm for 5 min and stored at -80°C.

*CgSUB2* was cloned in BamHI and SacI sites in pET-28a^+^ plasmid, and expressed in the *E*. *coli* BL21 (DE3) CodonPlus-RIL strain. Bacterial cells were grown for 3 h at 37°C, and *CgSUB2* expression was induced with 1 mM IPTG. After 3 h growth at 37°C, cells were collected, suspended in lysis buffer [Tris-HCl (50 mM; pH 8.0), NaCl (200 mM), imidazole (10 mM) and sarkosyl (1%)], and sonicated using Diagenode bioruptor plus. The recombinant CgSub2 protein carrying hexa-His epitope at the N-terminus was purified using Ni-NTA beads and polypropylene column (Thermo Fisher Scientific #R64050). After washing protein-bound beads with wash buffer [Tris-HCl (50 mM; pH 8.0), NaCl (150 mM) and imidazole (50 mM)], protein was eluted with the elution buffer [Tris-HCl (50 mM; pH 8.0), NaCl (150 mM) and imidazole (150 mM)]. Amicon Ultra centrifugal filter units (3 kDa cut off) were used to remove imidazole, and purified protein was stored at -80°C, after verifying protein purity on 12% SDS-PAGE.

For antibody generation in mice, purified CgHog1 or CgSub2 protein (100 μg), emulsified with complete Freud’s adjuvant were subcutaneously injected into 6–8 week-old BALB/c mice. After two booster doses at 15^th^ and 22^nd^ days, mice sera were collected and used to check the specificity of raised polyclonal antibodies by Western analysis. For anti-CgHog1 antibody, the mouse serum was pre-cleared with *Cghog1Δ* cell lysates (100 μg) for 12 h, prior to use. Anti-CgHog1 and anti-CgSub2 antibodies detected 51 kDa CgHog1 and 51 kDa CgSub2 protein bands in *wt* whole cell extracts, respectively.

### In vitro phosphorylation assay

CgSub2 phosphorylation was checked by in vitro kinase assay. Briefly, YNB-grown log phase *Cg* cells were left either untreated or treated with 1 mM FeCl_3_ for 2 h at 30°C. Cells were collected and lysed using glass bead in lysis buffer [Tris-HCl (50 mM; pH 7.5), NaCl (50 mM), EGTA (5 mM), MgCl_2,_ (5 mM), DTT (1 mM), triton X-100 (0.1%), and sodium orthovanadate (1 mM)]. Cell lysates were collected after centrifugation at 13000 rpm for 15 min at 4°C. For in vitro phosphorylation assay, the reaction mixture consisting of 200 μg cell lysate, 100 μg purified rCgSub2, and kinase buffer [Tris-HCl (50 mM; pH 7.5), NaCl (50 mM), EGTA (5 mM), MgCl_2,_ (20 mM), DTT (1 mM), and sodium orthovanadate (0.1 mM)] containing 10 μCi γ-^32^P ATP and 0.5 mM ATP, was prepared, and incubated for 1 h at 30°C. After adding SDS-PAGE sample buffer, samples were heated at 95°C for 5 min, and resolved on 10% SDS-PAGE gel. The gel was dried and exposed to the phosphor screen for 12–16 h, followed by screen scanning on Typhoon FLA 9500 biomolecular imager.

To detect the mobility shift of phosphorylated CgSub2 using Phos-tag gels, 100 μg purified rCgSub2 was incubated with 200 μg whole cell lysates and kinase buffer [Tris-HCl (50 mM; pH 7.5), NaCl (50 mM), EGTA (5 mM), MgCl_2,_ (20 mM), DTT (1 mM), and sodium orthovanadate (0.1 mM)] containing 0.5 mM ATP. After 1 h incubation at 30°C, SDS-PAGE sample buffer was added, and samples were heated at 95°C for 5 min, followed by sample resolution in a Phos-tag (50 μM; Phos-tag Acrylamide AAL-107, NARD)-containing 10% SDS-PAGE, and probing with anti-His and anti-CgSub2 antibodies.

### Human cell lines and culture conditions

The human kidney epithelial [A-498 (ATCC #HTB-44)], human stomach epithelial [AGS (ATCC #CRL-1739)] and human moncytic cell line [ATCC #TIB-202] were maintained in Minimum Essential Medium (MEM), Ham’s F-12 Medium (F-12K Medium), and RPMI medium, respectively, at 37°C and 5% CO_2_.

### Adherence measurement

A-498 and AGS cells were seeded at a seeding density of 2 × 10^5^ and 5 × 10^5^ cells, respectively, per well in a 24-well tissue culture plate and incubated at 37°C and 5% CO_2_ for 12–14 h, followed by cell fixation with 3.7% p-formaldehyde for 15 min. For adherence potential measurement, *Cg* strains were grown in YNB medium for 24 h at 30°C, followed by re-inoculation of 100 μl yeast culture in YNB medium containing 200 μCi S35(Met : Cys-65:25) [INVIVO PROTWIN label mix]. After 16 h growth at 30°C, radio-labelled *Cg* were harvested, and culture OD_600_ was normalized to 0.1. 200 μl S^35^-labelled *Cg* cell suspension (4 X 10^5^ *Cg* cells) was added to seeded epithelial cells, and incubated for 90 min at room temperature. After washing off non-adherent *Cg*, epithelial cells were lysed in 5% SDS, and radioactive counts (output values) in lysates were measured. The amount of radioactivity in labelled-*Cg* cell suspension was considered as ‘input value’. The percentage adherence was determined by dividing the output counts by input counts, and multiplying the obtained number by 100. To study the effect of high iron on adherence, *Cg* strains were grown in YNB medium containing ^35^S and 500 μM FeCl_3_ for 16 h, prior to incubation with fixed A-498 cells.

### Chromatin immunoprecipitation

Log-phase *Cg wt* cells were grown in YNB medium lacking or containing 1 mM FeCl_3_ for 2 h. *Cghog1Δ* and the conditional Cgsub2Δ* (*Cgsub2Δ/MET3Pro-CgSUB2*) mutants were grown in YNB and YNB medium containing 2 mM methionine and 2 mM cysteine, respectively. Both FeCl_3_-treated and untreated *Cg* cells were cross-linked with 1% formaldehyde for 20 min at 30°C, followed by reaction quenching with 125 mM glycine for 10 min at room temperature. Cells were harvested, and suspended in FA lysis buffer [EDTA (1 mM; pH 8.0), HEPES (50 mM; pH 7.5), NaCl (140 mM), sodium deoxycholate (0.1%), 1X protease inhibitor, and triton X-100 (1%)]. After cell lysis by bead-beating, lysates were spun down, and the supernatant was sonicated in cycles of 40 pulses (30 sec ON and 30 sec OFF) at the highest amplitude. The soluble fraction was collected after centrifugation at 15,000 rpm for 10 min, and 1/10th volume was stored as ‘Input fraction’. 3 mg protein lysate was immunoprecipitated with anti-CgSub2 antibody-conjugated protein-A Sepharose beads for 3 h at 4°C. Beads were sequentially washed with FA lysis buffer, FA lysis buffer containing 0.5 M NaCl, Wash buffer [Tris-HCl (100 mM; pH 8.0), LiCl (0.25 M), NP-40 (0.5%), deoxycholic acid (0.5%), and EDTA (1 mM)], and TE buffer. Finally, beads were suspended in elution buffer [Tris-HCl (50 mM; pH 8.0), EDTA (10 mM), and SDS (1%)] and subjected to de-crosslinking, along with the Input fraction, for overnight at 65°C. After proteinase K treatment for 1 h at 60°C, DNA was extracted, suspended in TE, treated with RNase for 1 h at 37°C, and used as a template for qRT-PCR to examine amplification of the *EPA1* promoter region.

### Other procedures

qRT-PCR and THP-1 macrophage infection were performed as described previously [4,23]. The freely available web tool cytoscape (https://cytoscape.org/) was used to depict the overlap among identified interacting partners of CgHog1.

### Statistical analysis

GraphPad Prism was used for statistical analysis with significance level p≤0.05. Data are presented as mean and standard error of the mean. The appropriate statistical tests used are indicated in figure legends.

## Supporting information

S1 Fig CgHog1 is required for *Cg* survival in the gastrointestinal candidiasis model. **A**. **Time-course analysis of *wt* (YRK20) and *Cghog1Δ* (YRK964) in YPD and YNB medium**. Overnight YPD and YNB medium-grown cultures were inoculated at an initial OD_600_ of 0.1 in YPD and YNB medium, respectively. Cultures were grown at 30°C and the absorbance was recorded at regular intervals till 48 h. Data represent mean ± SEM (n = 3–4). *, p < 0.0332; **, p < 0.0021; ***, p < 0.0002; ****, p < 0.0001, multiple t-test. **B**. 6–8 week-old female C57BL/6 mice were orally infected with 200 μl PBS suspension containing 2.5 X 10^8^ *wt* cells (1X inoculum), 2.5 X 10^8^ *Cghog1Δ* cells (1X inoculum) or 7.5 X 10^8^ *Cghog1Δ* cells (3X inoculum), using a 24-gauge feeding needle. At 1^st^ and 4^th^ day post-infection, mice were sacrificed and fungal load in indicated organs was determined. Circles and triangles represent CFUs in individual mouse organs at 1^st^ and 4^th^ day post-infection, respectively. Bars indicate the CFU geometric mean (n = 7–9). Black asterisks denote differences in organ CFUs between *wt* and *Cghog1Δ*-infected mice that were sacrificed on the same day. Olive asterisks denote organ CFU differences between 1^st^ and 4^th^ day-sacrificed mice, that were infected with the same *C*. *glabrata* strain. *, p ≤ 0.05; **, p ≤ 0.01; ***, p ≤ 0.001; ****, p ≤ 0.0001, Mann-Whitney U-test.
(TIF)

S2 Fig N-terminally SFB-tagged CgHog1 is functional. **A**. **Serial dilution spotting analysis showing that the N-terminally SFB-tagged CgHog1 rescues osmotic and high-iron stress sensitivity of *Cghog1Δ* mutant.** Overnight CAA medium-grown cultures were normalized to OD_600_ of 1, followed by spotting of 3 μl of three 10-fold serial dilutions on indicated medium. Sodium chloride (NaCl) and ferric chloride (FeCl_3_) were used at 1 M and 3 mM concentrations, respectively. V, empty vector. *wt/V*, *Cghog1Δ/V* and *Cghog1Δ/SFB-CgHOG1* strains correspond to YRK1057, YRK1564 and YRK1556 strains, respectively. **B**. **N-terminally SFB-tagged CgHog1 is hyperphosphorylated upon growth in high-iron medium.** *Cghog1Δ* carrying vector expressing either the SFB epitope (V; YRK1564) or SFB-CgHog1 (YRK1556) were grown overnight in CAA medium containing 50 μM BPS (extracellular iron chelator). These iron-starved cultures were grown in CAA medium lacking (Regular-iron; RI) or containing sodium ascorbate (1 mM) and ferrous ammonium sulfate (500 μM) (High-iron; HI) for 2 h. Whole cell lysates were prepared and resolved on 12% SDS-PAGE. Phosphorylated CgHog1 (CgHog1-P; 65 kDa), total CgHog1 (65 kDa) and CgGapdh (36 kDa) proteins were detected using anti-P-p38, anti-Flag and anti-Gapdh antibodies, respectively. The arrows and asterisks mark CgHog1 and non-specific protein bands, respectively.
(TIF)

S3 Fig CgSub2 is essential for cell growth. **A. Immunoblot analysis validating the specificity of the polyclonal antibody raised against CgSub2 protein.** Log-phase cells of *wt* (YRK20) and Cgsub2Δ* (YRK3294) -expressing *CgSUB2* from the methionine-repressible *CgMET3* promoter, were lysed using glass beads. Whole cell lysates (100 μg) were resolved on 12% SDS-PAGE, followed by immunoblotting with anti-CgSub2 and anti-Gapdh antibodies. CgSub2 and CgSub2-SFB corresponded to 51 kDa and 65 kDa sizes, respectively. **B. Immunoblot analysis illustrating the specificity of the polyclonal antibody raised against CgHog1 protein.** Log-phase cells of *wt* (YRK20) and *Cghog1Δ* (YRK964) were lysed using glass beads. Whole cell lysates (100 μg) were resolved on 12% SDS-PAGE, followed by immunoblotting with anti-CgHog1 and anti-Gapdh antibodies. CgHog1 band corresponded to 51 kDa size. **C. Phosphorylated CgHog1 interacts with CgSub2.** Log-phase *wt* (YRK20) cells were grown in YNB medium lacking or containing 1 mM ferric chloride for 2 h. Cells were lysed using glass beads and cell lysates were incubated with anti-P-p38 antibody for 12 h at 4° C. Immunoprecipitated (IP) and cell lysate (Input) samples were resolved on 10% SDS-PAGE, followed by probing with anti-P-p38, anti-CgSub2 and anti-Gapdh antibodies to detect phosphorylated CgHog1, CgSub2 and CgGapdh, respectively. D. Serial dilution spotting analysis illustrating that the Cgsub2Δ* (YRK3294) strain could not grow in YNB medium containing 2 mM methionine (Met) and 2 mM cysteine (Cys). Notably, *CgSUB2* expression is abated in the presence of methionine and cysteine, as *CgSUB2* is being expressed from the methionine-repressible *CgMET3* promoter. Plates were imaged after 2 days of growth at 30°C. E. Time-course analysis of Cgsub2Δ* (YRK3294) in YNB medium containing 2 mM methionine (Met) and 2 mM cysteine (Cys). Overnight YNB medium-grown cultures were inoculated at an initial OD_600_ of 0.2 in YNB lacking or containing 2 mM methionine and 2 mM cysteine. Cultures were grown at 30°C and absorbance was recorded at regular intervals till 48 h. Data represent mean ± SEM (n = 3–4). *, p < 0.0332; **, p < 0.0021; ****, p < 0.0001, multiple t-test. **F. Immunoblot showing CgSub2-SFB levels.** Overnight YNB medium-grown Cgsub2Δ* (YRK3294) cells were inoculated in YNB medium lacking or containing 2 mM methionine (Met) and 2 mM cysteine (Cys). YNB medium-grown *wt* cells were used as control. At indicated time points, cells were collected, lysed and whole cell lysates (100 μg) were resolved on 12% SDS-PAGE, followed by immunoblotting with anti-CgSub2 and anti-Gapdh antibodies. **G. Intracellular iron levels in indicated strains.** *wt* (YRK20) and *Cghog1Δ* (YRK964) strains were grown to log-phase in YNB medium lacking or containing methionine (2 mM) and cysteine (2 mM). Data represent mean ± SEM (n = 3). Black and red asterisks denote fold-differences in iron content between *Cghog1Δ* and *wt* (taken as 1.0) grown in the same medium, respectively. *, p ≤ 0.05; two-tailed Student’s *t* test. **H. Immunoblot showing CgSub2-SFB levels upon *CgSUB2* overexpression.** Overnight YNB medium-grown *wt* (YRK20) and *wt* cells overexpressing *CgSUB2-SFB* from *PDC1* promoter (*wt/CgSUB2-SFB*; YRK2803) were collected. As a control, Cgsub2Δ* (YRK3294) cells that were grown overnight in YNB medium lacking or containing 2 mM methionine and 2 mM cysteine were collected. Whole cell lysates were prepared and 100 μg samples were resolved on 12% SDS-PAGE, followed by immunoblotting with anti-CgSub2 and anti-Gapdh antibodies. Anti-Sub2 antibody detected both endogenous (51 kDa) and SFB-tagged CgSub2 (65 kDa) protein. A faint signal corresponding to CgSub2-SFB in Cgsub2Δ* could be due to the leaky expression of *CgSUB2-SFB* from the *MET3* promoter.
(TIF)

S4 Fig CgHog1 kinase activity is required for CgSub2 phosphorylation. **A. Multiple amino acid sequence alignment of Hog1 homologs from *Homo sapiens* (*Hs*), *Candida albicans* (*Ca*), *Schizosaccharomyces pombe* (*Sp*), *Saccharomyces cerevisiae* (*Sc*) and *Candida glabrata* (*Cg*) showing conserved catalytic lysine and phosphorylatable threonine and tyrosine residues.** Clustal Omega (https://www.ebi.ac.uk/Tools/msa/clustalo/) was used to align sequences retrieved from the Uniprot database (https://www.uniprot.org/) for each protein. The black and orange arrows mark the conserved catalytic and phosphorylatable residues, respectively. **B. A representative Phos-tag gel illustrating CgSub2 mobility shift upon incubation with cell lysates of the *wt* (YRK20) strain grown in YNB medium containing 1 mM ferric chloride for 2 h.** Reaction mixtures of the in vitro phosphorylation assay were resolved on Phos-tag/SDS-PAGE (10%) and SDS-PAGE (10%) gels, and immunoblotted. **C. A representative autoradiograph showing phosphorylated CgSub2.** Log-phase *Cghog1Δ* cultures expressing wild-type (YRK1047), catalytically-dead (CgHog1^*K52A*^; YRK2127), non-phosphorylatable (CgHog1^*T174A/Y176F*^; YRK2129) CgHog1 or empty vector (V; used as control; YRK1166) were grown in YNB medium containing FeCl_3_ (1 mM) for 2 h. Whole cell lysates were prepared using glass beads, and 200 μg cell lysates were incubated with 100 μg *E*. *coli-*purified rCgSub2, 0.5 mM ATP and 10 μCi γ-^32^P-ATP at 30°C. Samples were resolved on 10% SDS-PAGE, and analyzed by autoradiography. The first lane contained all reagents but for the cell lysate. **D. Adherence analysis of indicated S**^**35**^**-labelled *Cg* cells to A-498 kidney epithelial cells.** Data represent mean ± SD; (n = 2). Black and red asterisks represent changes in the percentage adherence between *wt* (YRK20) and mutants, and *Cghog1Δ* (YRK964) and *Cghog1Δepa1Δ* (YRK5397), respectively. *p ≤ 0.05; **p ≤ 0.01, unpaired two-tailed Student’s *t*-test. **E. Adherence analysis of indicated *Cg* strains to A-498 cells via CFU-based assay.** Overnight-grown *wt* (YRK20) and Cgsub2Δ* (YRK3294) strains were sub-cultured in YNB medium lacking or containing cold methionine (2 mM) and cysteine (2 mM). *Cg* cells were collected and incubated with p-formaldehyde-fixed A-498 cells at 2:1 MoI. After 2 h incubation at room temperature, A-498 epithelial cells were washed with PBS and lysed in 1 ml PBS. The lysate was plated on YNB medium, and *Cg* colonies (Output) appeared were counted. To determine % adherence, the output CFUs were divided by input CFUs (Number of *Cg* cells infected to A-498 cells), and the number was multiplied by 100. Data represent mean ± SEM (n = 3). Black and red asterisks denote statistically significant differences in the percentage adherence between YNB-grown *wt* and methionine and cysteine-containing YNB-grown Cgsub2Δ*, and YNB-grown Cgsub2Δ* and methionine and cysteine-containing YNB-grown Cgsub2Δ*, respectively. ***, p ≤ 0.001, unpaired two-tailed Student’s *t*-test. **F. Adherence analysis of indicated S**^**35**^**-labelled *Cg* cells to AGS stomach epithelial cells.** Data (mean ± SD; n = 2–3) represent changes in the percentage adherence in indicated strains, compared to *wt* cells. *p ≤ 0.05; **p ≤ 0.01, unpaired two-tailed Student’s *t*-test.
(TIF)

S5 Fig CgSub2 is required for *Cg* survival in the gastrointestinal candidiasis model. *wt* (YRK20), Cgsub2Δ* (YRK3294) and *wt/CgSUB2* (YRK2803) strains were grown overnight in YNB, methionine and cysteine-containing YNB and YNB media, respectively. Cells were collected, washed and suspended in PBS. Groups of 6–8 week-old, female C57BL/6 mice were orally infected with 2.5 X 10^8^ cells (200 μl PBS suspension) of *wt*, Cgsub2Δ* or *wt/CgSUB2* strain, using a 24-gauge feeding needle. At 1^st^ and 4^th^ day post-infection, mice were sacrificed and fungal load in indicated organs was determined. Circles and triangles represent CFUs in individual mouse organs at 1^st^ and 4^th^ day post-infection, respectively. Bars indicate the CFU geometric mean (n = 6–10). Black asterisks denote differences in organ CFUs between *wt* and indicated strain-infected mice that were sacrificed on the same day. Olive asterisks denote organ CFU differences between 1^st^ and 4^th^ day-sacrificed mice, that were infected with the same *C*. *glabrata* strain. *, p ≤ 0.05; **, p ≤ 0.01; ***, p ≤ 0.001; ****, p ≤ 0.0001, Mann-Whitney U-test.
(TIF)

S6 Fig Multiple amino acid sequence alignment of CgHtz1 (encoded by *CAGL0E02315g* ORF) and the canonical histone H2A (CgHta) that is endoded by two ORFs, *CAGL0K11440g* (*CgHTA1*), and *CAGL0C04411g* (*CgHTA2*). The Clustal Omega multiple sequence alignment tool (https://www.ebi.ac.uk/Tools/msa/clustalo/) was used to align sequences retrieved from the CGD database (http://www.candidagenome.org/). Black asterisks indicate identical amino acids.
(TIF)

S7 Fig CgHtz1 levels are reduced under high-iron conditions. **A. Serial dilution spotting analysis illustrating sensitivity of indicated strains to DNA damage [MMS (methyl methanesulfonate; 0.04%)] and oxidative [Hydrogen peroxide (H**_**2**_**O**_**2**_**; 20 mM)] stresses.** Growth was recorded after 2–3 days of incubation at 30°C. *wt*, *Cghog1Δ*, *Cghog1Δ+CgHOG1*, *Cghtz1Δ*, *Cghtz1Δ+CgHTZ1*, *Cghog1Δhtz1Δ*, *Cghog1Δhtz1Δ+CgHTZ1*, *Cghog1Δhtz1Δ+CgHOG1* strains correspond to YRK20, YRK964, YRK6290, YRK4392, YRK4737, YRK4390, YRK5123 and YRK6292 strains, respectively. **B. Intracellular replication analysis.** Human THP-1 monocytic cells were treated with PMA (phorbol 12-myristate 13-acetate; 16 nM) for 12 h, followed by recovery in the fresh RPMI medium for 12 h. Overnight YPD-medium-grown *Cg* strains were infected to differentiated THP-1 macrophages at MoI (multiplicity of infection) of 1:10. After 2 h incubation, non-internalized *Cg* cells were washed off with PBS, and the infection was continued for another 22 h. Infected macrophages were lysed in water at 2 h and 24 h post infection, and lysates were diluted in PBS and plated on YPD medium. After 1–2 days of incubation at 30°C, *Cg* colonies were counted, and the number was multiplied by the appropriate dilution factor. Fold replication (mean ± SEM; n = 3) represents the ratio of the number of intracellular *Cg* cells at 24 h to that at 2 h post infection for each strain. ***, p ≤ 0.001; unpaired two-tailed Student’s t test. *wt*, *Cghtz1Δ* and *Cghtz1Δ+CgHTZ1* strains correspond to YRK20, YRK4392 and YRK4737 strains, respectively. **C. CgHog1 does not interact with CgHtz1.** Lysates (6 mg) of indicated, YNB medium-grown strains were incubated with anti-CgHog1 antibody for 12 h. Immunoprecipitated (IP) and cell lysate (Input) samples were resolved on 15% SDS-PAGE, followed by probing with anti-CgHog1, anti-Htz1 and anti-Gapdh antibodies. CgGapdh was used as loading control. *wt*, *Cghog1Δ* and *Cghtz1Δ* strains correspond to YRK20, YRK964 and YRK4392 strains, respectively. **D. Immunoblot showing phosphorylated CgHog1 levels.** Lysates (100 μg) of indicated strains were resolved on 12% SDS-PAGE, followed by immunoblotting with anti-P-p38 (Hog1-P) antibody, anti-CgHog1 and anti-Gapdh antibodies to detect phosphorylated CgHog1, total CgHog1 and CgGapdh proteins, respectively. CgGapdh was used as loading control. *wt*, *Cghog1Δ*, Cgsub2Δ* and *Cghtz1Δ* strains correspond to YRK20, YRK964, YRK3294 and YRK4392 strains, respectively. The Cgsub2Δ* strain was grown in methionine and cysteine-containing YNB medium, while other strains were grown in YNB medium. **E. Immunoblot analysis showing diminished CgHtz1 levels under high-iron conditions.** Log-phase *wt* (YRK20) cells were grown in YNB medium lacking or containing 1 mM and 2 mM ferric chloride for 2 h. Cells were lysed using glass beads, and whole cell lysates (100 μg) were resolved on 15% SDS-PAGE, followed by immunoblotting with anti-P-p38 (CgHog11-P) antibody, anti-Htz1 and anti-Gapdh antibodies to detect phosphorylated CgHog1, CgHtz1 and CgGapdh proteins, respectively. CgGapdh was used as loading control. CgHtz1 signal was normalized with the corresponding CgGapdh signal. Fold-change in CgHtz1 levels in ferric chloride-containing medium, compared to YNB medium (taken as 1.0), is plotted on the right side of the blot panel. **, *p* ≤ 0.01, paired two-tailed Student’s t-test. **F. Immunoprecipitation analysis illustrating reduced CgSub2-CgHtz1 interaction under high-iron conditions**. Lysates (15 mg) of *wt* (YRK20) cells, that were grown in YNB medium lacking or containing 1 mM ferric chloride for 2 h, were incubated with anti-CgSub2 antibody. After 12 h, immunoprecipitated (IP) and cell lysate (Input) samples were resolved on 15% SDS-PAGE and immunoblotted. Fold-decrease in CgSub2-CgHtz1 interaction (mean ± SD; n = 2) in ferric chloride-grown *wt*, compared to YNB-grown *wt* cells (taken as 1.0), is shown underneath the blot. *Cghog1Δ* cell lysates, used as control, showed elevated CgSub2-CgHtz1 interaction. *, p ≤ 0.05, paired two-tailed Student’s *t* test.
(TIF)

S8 Fig CgHog1 and CgHtz1 negatively and positively regulate *EPA1* expression, respectively. **A. Immunoblot showing CgHtz1 levels upon *CgHTZ1* overexpression.** Overnight CAA medium-grown *wt* (YRK20), *Cghtz1Δ* (YRK4392) and *wt* cells overexpressing *CgHTZ1* from *PDC1* promoter (*wt/HTZ1*; YRK4729) were collected. Whole cell lysates were prepared and 100 μg samples were resolved on 15% SDS-PAGE, followed by immunoblotting with anti-Htz1 and anti-Gapdh antibodies. Data (mean ± SEM; n = 3) represent fold-increase in CgHtz1 levels in *wt/HTZ1*, compared to *wt* (taken as 1.0). **, *p* ≤ 0.01, paired two-tailed Student’s *t*-test. **B. qRT-PCR-based *EPA1* expression analysis.** Data (mean ± SEM, n = 3) were normalized against *CgACT1* mRNA control, and represent fold change in *EPA1* transcript levels in log-phase *Cghtz1Δ* (YRK4392) and *Cghog1Δ* (taken as control; YRK964) cells, compared to log-phase *wt* (YRK20) cells (considered as 1.0). *p ≤ 0.05; ***, p ≤ 0.001, paired two-tailed Student’s *t*-test.
(TIF)

S9 Fig CgSub2 is phosphorylated at four amino acid residues. **A. A schematic illustration of domains predicted in 439 amino acid-long CgSub2 protein using ExPASy PROSITE proteomics analysis server** (https://prosite.expasy.org/prosite.html). Q_MOTIF (55–83 aa): DEAD-box RNA helicase Q motif, HELICASE_ATP_BIND_1 (86–261 aa): Superfamilies 1 and 2 helicase ATP-binding type-1 domain, and HELICASE_CTER (273–434 aa): Superfamilies 1 and 2 helicase C-terminal domain. The diagram is not drawn to scale. **B. Multiple amino acid sequence alignment of *Homo sapiens* (*Hs*; DX39B), *Saccharomyces cerevisiae* (*Sc*; Sub2), *Drosophila melanogaster* (*Dm*; DX39B) and *Candida glabrata* (*Cg*; Sub2) Sub2 proteins showing conserved identified four phosphorylatable residues (Thr-176, Ser-200, Thr-230 and Thr-395) in CgSub2.** Clustal Omega (https://www.ebi.ac.uk/Tools/msa/clustalo/) was used to align sequences retrieved from the Uniprot database (https://www.uniprot.org/) for each protein. The brown and black arrows mark the conserved residues between *Cg* and *Sc* Sub2, and Sub2 in all four organisms, respectively. **C. Adherence analysis of S**^**35**^**-labelled *wt* cells, that were overexpressing CgSub2 variants with indicated serine and threonine residues mutated to alanine, to A-498 cells.** Overexpression of both wild-type and mutated CgSub2 rendered *Cg* cells hyperadherent. Data represent mean ± SEM (n = 3). **p ≤ 0.01; ***p ≤ 0.001, unpaired two-tailed Student’s *t*-test. *wt*, *wt/CgSUB2*, *wt/CgSUB2*^*T176A*^, *wt/CgSUB2*^*S200A*^, *wt/CgSUB2*^*T230A*^ and *wt/CgSUB2*^*T395A*^ strains correspond to YRK20, YRK2803, YRK5167, YRK4640, YRK5121 and YRK5171 strains, respectively. **D. qRT-PCR-based measurement of *EPA3* and *EPA6* transcript levels.** *wt* (YRK20), Cgsub2Δ* (YRK3294) and *wt/CgSUB2* (YRK2803) strains were grown to log-phase in YNB, methionine and cysteine-containing YNB and YNB medium, respectively. Total RNA was isolated, and 500 ng RNA was used for cDNA synthesis, followed by real-time quantitative PCR amplification. Transcript levels were quantified using the 2-^ΔΔ^C_t_ method. Data (mean ± SD, n = 2–3) were normalized against *CgACT1* mRNA control, and represent fold change in *EPA3* and *EPA6* expression in indicated strains compared to *wt* (taken as 1.0). **p ≤ 0.01, paired two-tailed Student’s *t*-test. **E. Serial dilution spotting analysis showing sensitivity of *Cgino80****Δ* **(YRK2161) and *Cgswr1****Δ* **(YRK2166) mutants to ferric chloride (4 mM).** Plates were imaged after 2 days of growth at 30°C.
(TIF)

S1 Table CgHog1-interactome analysis. CgHog1-interacting proteins identified under low-iron (S1A Table), regular-iron (S1B Table) and high-iron (S1C Table) conditions.
(XLSX)

S2 Table Enriched gene ontology (GO) terms for biological process (BP), cellular component (CC) and molecular function (MF) categories, as determined by the FungiFun tool, in CgHog1-interacting proteins identified under regular, high and low-iron conditions. (XLSX)

S3 Table A list of CgSub2-interacting proteins in wild-type and *Cghog1Δ* strains, as identified by mass spectrometry analysis. (XLSX)

S4 Table Enriched gene ontology (GO) terms for biological process (BP), cellular component (CC) and molecular function (MF) categories, as determined by the FungiFun tool, in common and unique CgSub2-interacting proteins in wild-type and *Cghog1Δ* strains. (XLSX)

S5 Table List of strains used in the study. (XLSX)

S6 Table List of plasmids used in the study. (XLSX)

S7 Table List of primers used in the study. (XLSX)

S8 Table List of antibodies used in the study. (XLSX)

S9 Table Raw numerical data underlying plotted graphs. (XLSX)
