# Supplementary figures and images for: The Hog1 MAPK substrate governs Candida glabrata-epithelial cell adhesion via the histone H2A variant
Source: PLoS Genet. 2024 May 14;20(5):e1011281. doi: 10.1371/journal.pgen.1011281 (PMC11125552; doi:10.1371/journal.pgen.1011281)

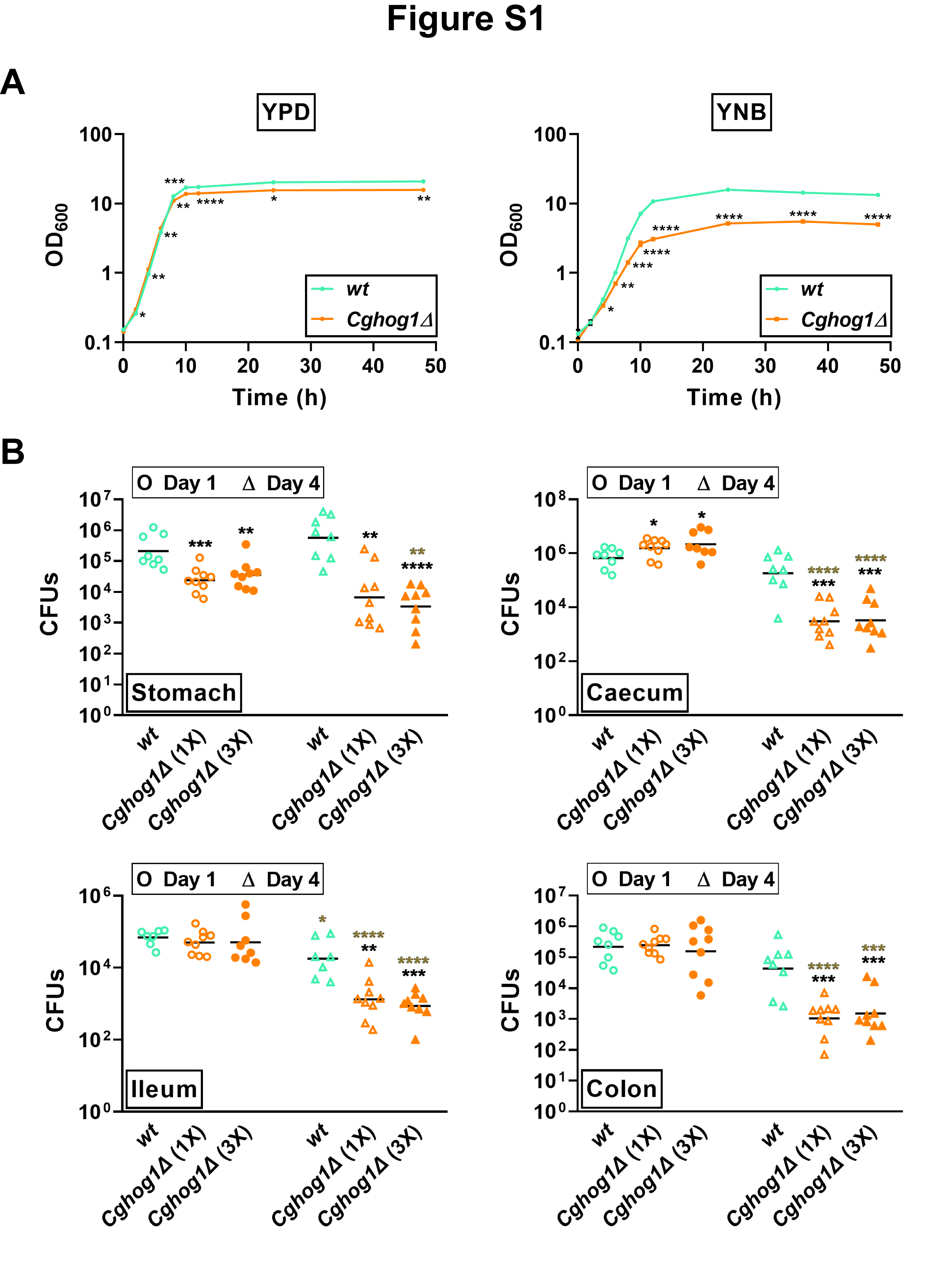

Supplement: S1 Fig — A. Time-course analysis of wt (YRK20) and Cghog1Δ (YRK964) in YPD and YNB medium. Overnight YPD and YNB medium-grown cultures were inoculated at an initial OD600 of 0.1 in YPD and YNB medium, respectively. Cultures were grown at 30°C and the absorbance was recorded at regular intervals till 48 h. Data represent mean ± SEM (n = 3–4). *, p < 0.0332; **, p < 0.0021; ***, p < 0.0002; ****, p < 0.0001, multiple t-test. B. 6–8 week-old female C57BL/6 mice were orally infected with 200 μl PBS suspension containing 2.5 X 108 wt cells (1X inoculum), 2.5 X 108 Cghog1Δ cells (1X inoculum) or 7.5 X 108 Cghog1Δ cells (3X inoculum), using a 24-gauge feeding needle. At 1st and 4th day post-infection, mice were sacrificed and fungal load in indicated organs was determined. Circles and triangles represent CFUs in individual mouse organs at 1st and 4th day post-infection, respectively. Bars indicate the CFU geometric mean (n = 7–9). Black asterisks denote differences in organ CFUs between wt and Cghog1Δ-infected mice that were sacrificed on the same day. Olive asterisks denote organ CFU differences between 1st and 4th day-sacrificed mice, that were infected with the same C. glabrata strain. *, p ≤ 0.05; **, p ≤ 0.01; ***, p ≤ 0.001; ****, p ≤ 0.0001, Mann-Whitney U-test. (TIF) [file pgen.1011281.s001.tif]

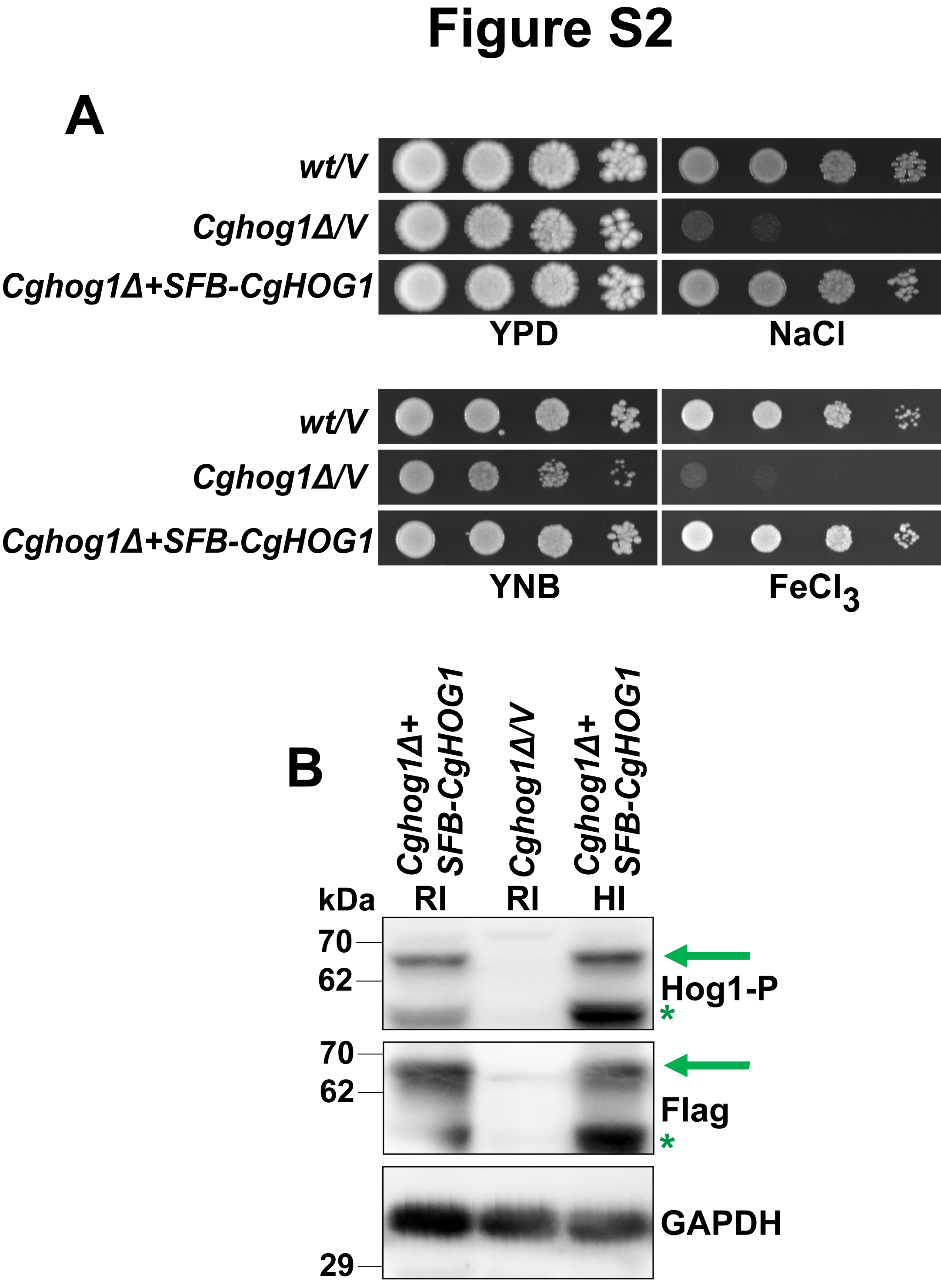

Supplement: S2 Fig — A. Serial dilution spotting analysis showing that the N-terminally SFB-tagged CgHog1 rescues osmotic and high-iron stress sensitivity of Cghog1Δ mutant. Overnight CAA medium-grown cultures were normalized to OD600 of 1, followed by spotting of 3 μl of three 10-fold serial dilutions on indicated medium. Sodium chloride (NaCl) and ferric chloride (FeCl3) were used at 1 M and 3 mM concentrations, respectively. V, empty vector. wt/V, Cghog1Δ/V and Cghog1Δ/SFB-CgHOG1 strains correspond to YRK1057, YRK1564 and YRK1556 strains, respectively. B. N-terminally SFB-tagged CgHog1 is hyperphosphorylated upon growth in high-iron medium. Cghog1Δ carrying vector expressing either the SFB epitope (V; YRK1564) or SFB-CgHog1 (YRK1556) were grown overnight in CAA medium containing 50 μM BPS (extracellular iron chelator). These iron-starved cultures were grown in CAA medium lacking (Regular-iron; RI) or containing sodium ascorbate (1 mM) and ferrous ammonium sulfate (500 μM) (High-iron; HI) for 2 h. Whole cell lysates were prepared and resolved on 12% SDS-PAGE. Phosphorylated CgHog1 (CgHog1-P; 65 kDa), total CgHog1 (65 kDa) and CgGapdh (36 kDa) proteins were detected using anti-P-p38, anti-Flag and anti-Gapdh antibodies, respectively. The arrows and asterisks mark CgHog1 and non-specific protein bands, respectively. (TIF) [file pgen.1011281.s002.tif]

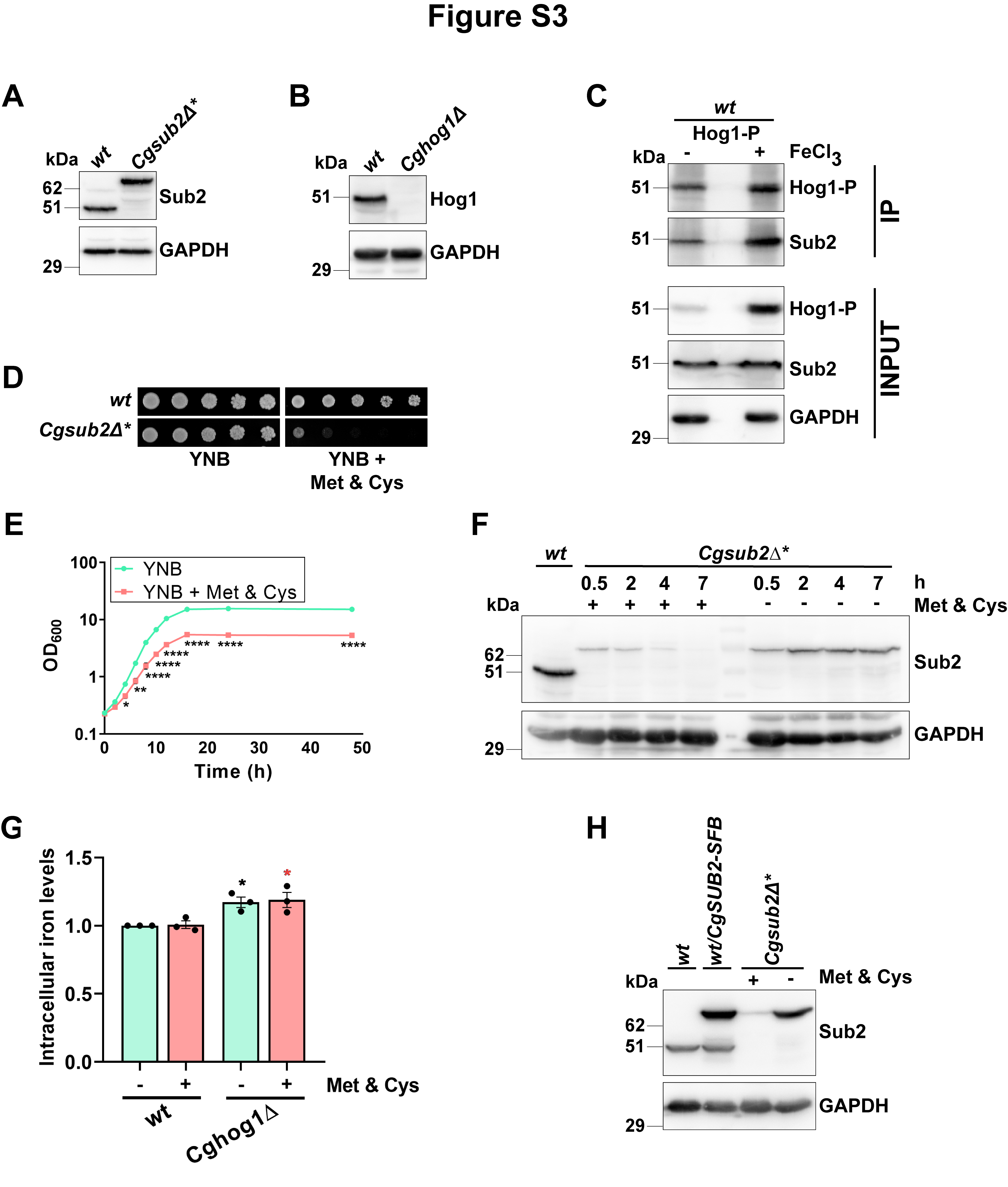

Supplement: S3 Fig — A. Immunoblot analysis validating the specificity of the polyclonal antibody raised against CgSub2 protein. Log-phase cells of wt (YRK20) and Cgsub2Δ* (YRK3294) -expressing CgSUB2 from the methionine-repressible CgMET3 promoter, were lysed using glass beads. Whole cell lysates (100 μg) were resolved on 12% SDS-PAGE, followed by immunoblotting with anti-CgSub2 and anti-Gapdh antibodies. CgSub2 and CgSub2-SFB corresponded to 51 kDa and 65 kDa sizes, respectively. B. Immunoblot analysis illustrating the specificity of the polyclonal antibody raised against CgHog1 protein. Log-phase cells of wt (YRK20) and Cghog1Δ (YRK964) were lysed using glass beads. Whole cell lysates (100 μg) were resolved on 12% SDS-PAGE, followed by immunoblotting with anti-CgHog1 and anti-Gapdh antibodies. CgHog1 band corresponded to 51 kDa size. C. Phosphorylated CgHog1 interacts with CgSub2. Log-phase wt (YRK20) cells were grown in YNB medium lacking or containing 1 mM ferric chloride for 2 h. Cells were lysed using glass beads and cell lysates were incubated with anti-P-p38 antibody for 12 h at 4° C. Immunoprecipitated (IP) and cell lysate (Input) samples were resolved on 10% SDS-PAGE, followed by probing with anti-P-p38, anti-CgSub2 and anti-Gapdh antibodies to detect phosphorylated CgHog1, CgSub2 and CgGapdh, respectively. D. Serial dilution spotting analysis illustrating that the Cgsub2Δ* (YRK3294) strain could not grow in YNB medium containing 2 mM methionine (Met) and 2 mM cysteine (Cys). Notably, CgSUB2 expression is abated in the presence of methionine and cysteine, as CgSUB2 is being expressed from the methionine-repressible CgMET3 promoter. Plates were imaged after 2 days of growth at 30°C. E. Time-course analysis of Cgsub2Δ* (YRK3294) in YNB medium containing 2 mM methionine (Met) and 2 mM cysteine (Cys). Overnight YNB medium-grown cultures were inoculated at an initial OD600 of 0.2 in YNB lacking or containing 2 mM methionine and 2 mM cysteine. Cultures were grown at 30°C and absorb [file pgen.1011281.s003.tif]

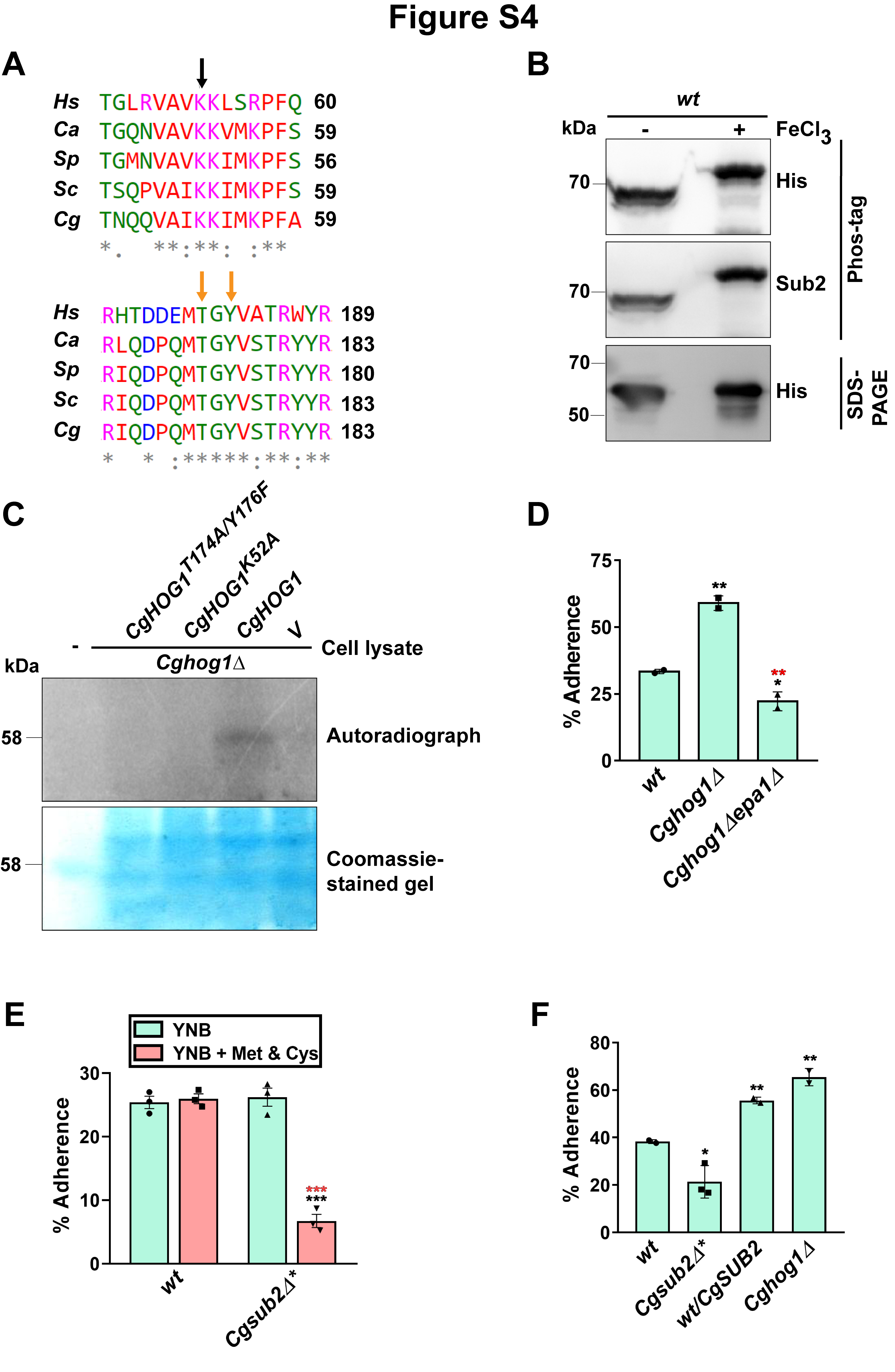

Supplement: S4 Fig — A. Multiple amino acid sequence alignment of Hog1 homologs from Homo sapiens (Hs), Candida albicans (Ca), Schizosaccharomyces pombe (Sp), Saccharomyces cerevisiae (Sc) and Candida glabrata (Cg) showing conserved catalytic lysine and phosphorylatable threonine and tyrosine residues. Clustal Omega (https://www.ebi.ac.uk/Tools/msa/clustalo/) was used to align sequences retrieved from the Uniprot database (https://www.uniprot.org/) for each protein. The black and orange arrows mark the conserved catalytic and phosphorylatable residues, respectively. B. A representative Phos-tag gel illustrating CgSub2 mobility shift upon incubation with cell lysates of the wt (YRK20) strain grown in YNB medium containing 1 mM ferric chloride for 2 h. Reaction mixtures of the in vitro phosphorylation assay were resolved on Phos-tag/SDS-PAGE (10%) and SDS-PAGE (10%) gels, and immunoblotted. C. A representative autoradiograph showing phosphorylated CgSub2. Log-phase Cghog1Δ cultures expressing wild-type (YRK1047), catalytically-dead (CgHog1K52A; YRK2127), non-phosphorylatable (CgHog1T174A/Y176F; YRK2129) CgHog1 or empty vector (V; used as control; YRK1166) were grown in YNB medium containing FeCl3 (1 mM) for 2 h. Whole cell lysates were prepared using glass beads, and 200 μg cell lysates were incubated with 100 μg E. coli-purified rCgSub2, 0.5 mM ATP and 10 μCi γ-32P-ATP at 30°C. Samples were resolved on 10% SDS-PAGE, and analyzed by autoradiography. The first lane contained all reagents but for the cell lysate. D. Adherence analysis of indicated S35-labelled Cg cells to A-498 kidney epithelial cells. Data represent mean ± SD; (n = 2). Black and red asterisks represent changes in the percentage adherence between wt (YRK20) and mutants, and Cghog1Δ (YRK964) and Cghog1Δepa1Δ (YRK5397), respectively. *p ≤ 0.05; **p ≤ 0.01, unpaired two-tailed Student’s t-test. E. Adherence analysis of indicated Cg strains to A-498 cells via CFU-based assay. Overnight-grown wt (YRK20) and Cgsub2Δ* (YRK3294) st [file pgen.1011281.s004.tif]

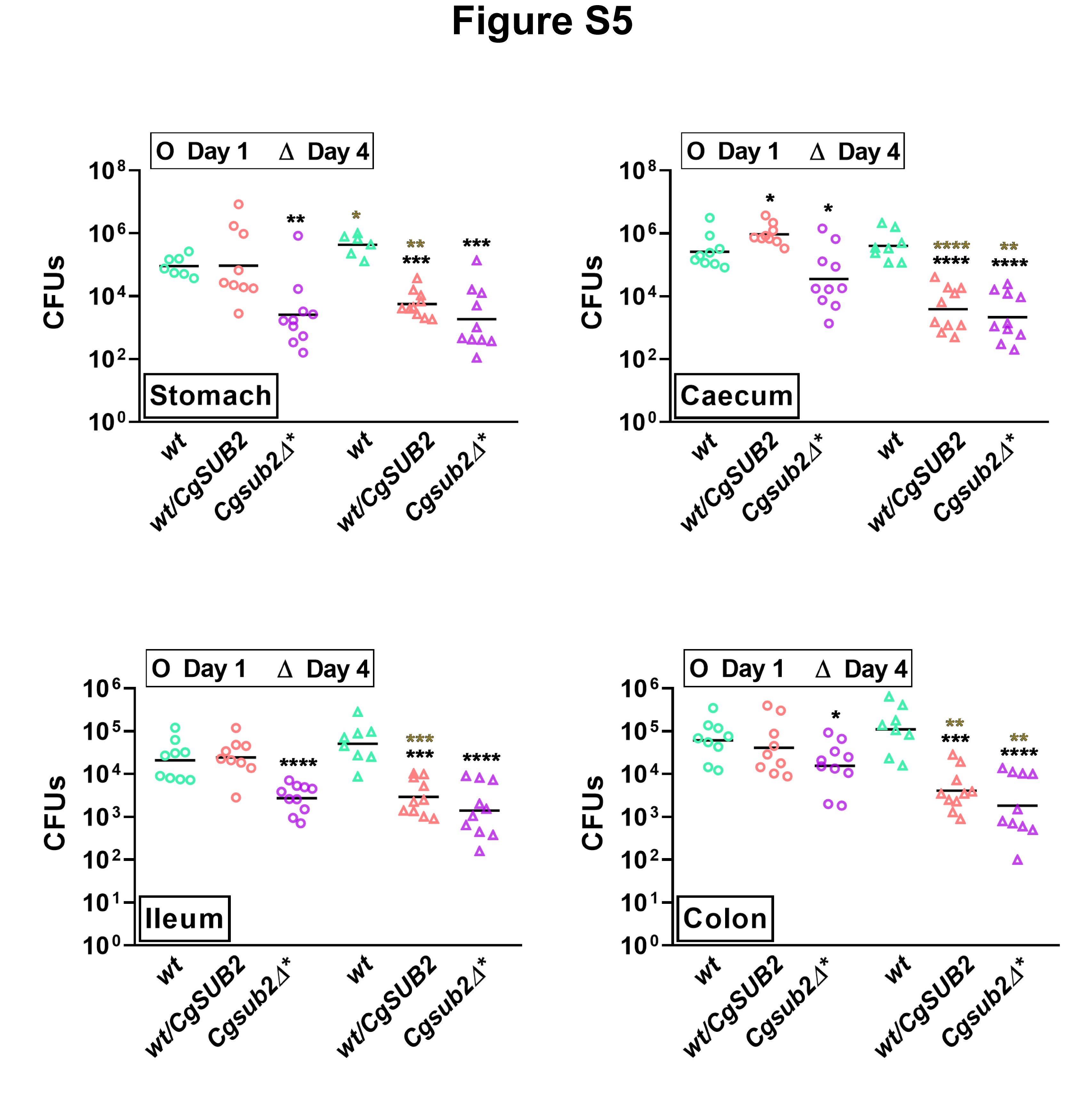

Supplement: S5 Fig — wt (YRK20), Cgsub2Δ* (YRK3294) and wt/CgSUB2 (YRK2803) strains were grown overnight in YNB, methionine and cysteine-containing YNB and YNB media, respectively. Cells were collected, washed and suspended in PBS. Groups of 6–8 week-old, female C57BL/6 mice were orally infected with 2.5 X 108 cells (200 μl PBS suspension) of wt, Cgsub2Δ* or wt/CgSUB2 strain, using a 24-gauge feeding needle. At 1st and 4th day post-infection, mice were sacrificed and fungal load in indicated organs was determined. Circles and triangles represent CFUs in individual mouse organs at 1st and 4th day post-infection, respectively. Bars indicate the CFU geometric mean (n = 6–10). Black asterisks denote differences in organ CFUs between wt and indicated strain-infected mice that were sacrificed on the same day. Olive asterisks denote organ CFU differences between 1st and 4th day-sacrificed mice, that were infected with the same C. glabrata strain. *, p ≤ 0.05; **, p ≤ 0.01; ***, p ≤ 0.001; ****, p ≤ 0.0001, Mann-Whitney U-test. (TIF) [file pgen.1011281.s005.tif]

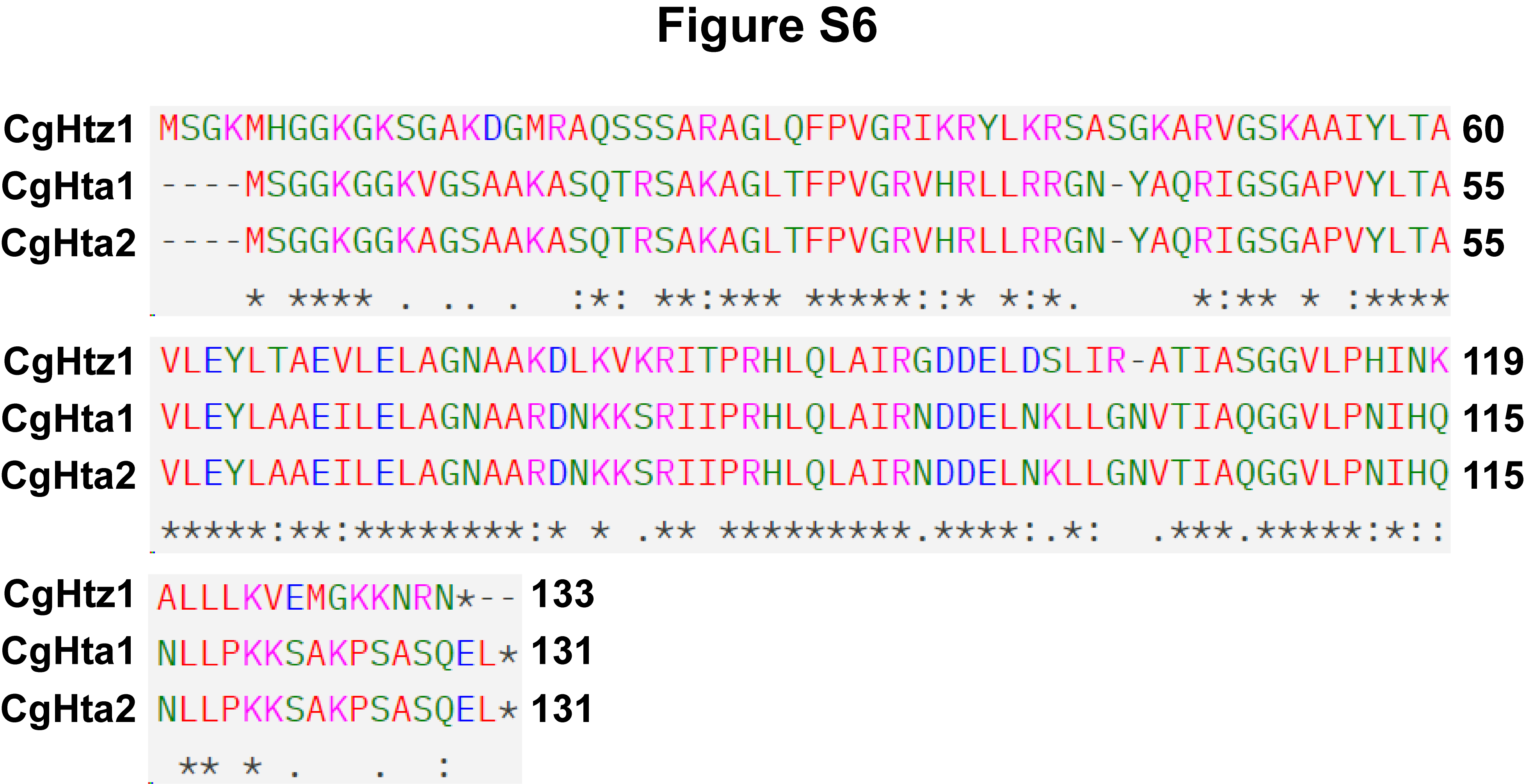

Supplement: S6 Fig — The Clustal Omega multiple sequence alignment tool (https://www.ebi.ac.uk/Tools/msa/clustalo/) was used to align sequences retrieved from the CGD database (http://www.candidagenome.org/). Black asterisks indicate identical amino acids. (TIF) [file pgen.1011281.s006.tif]

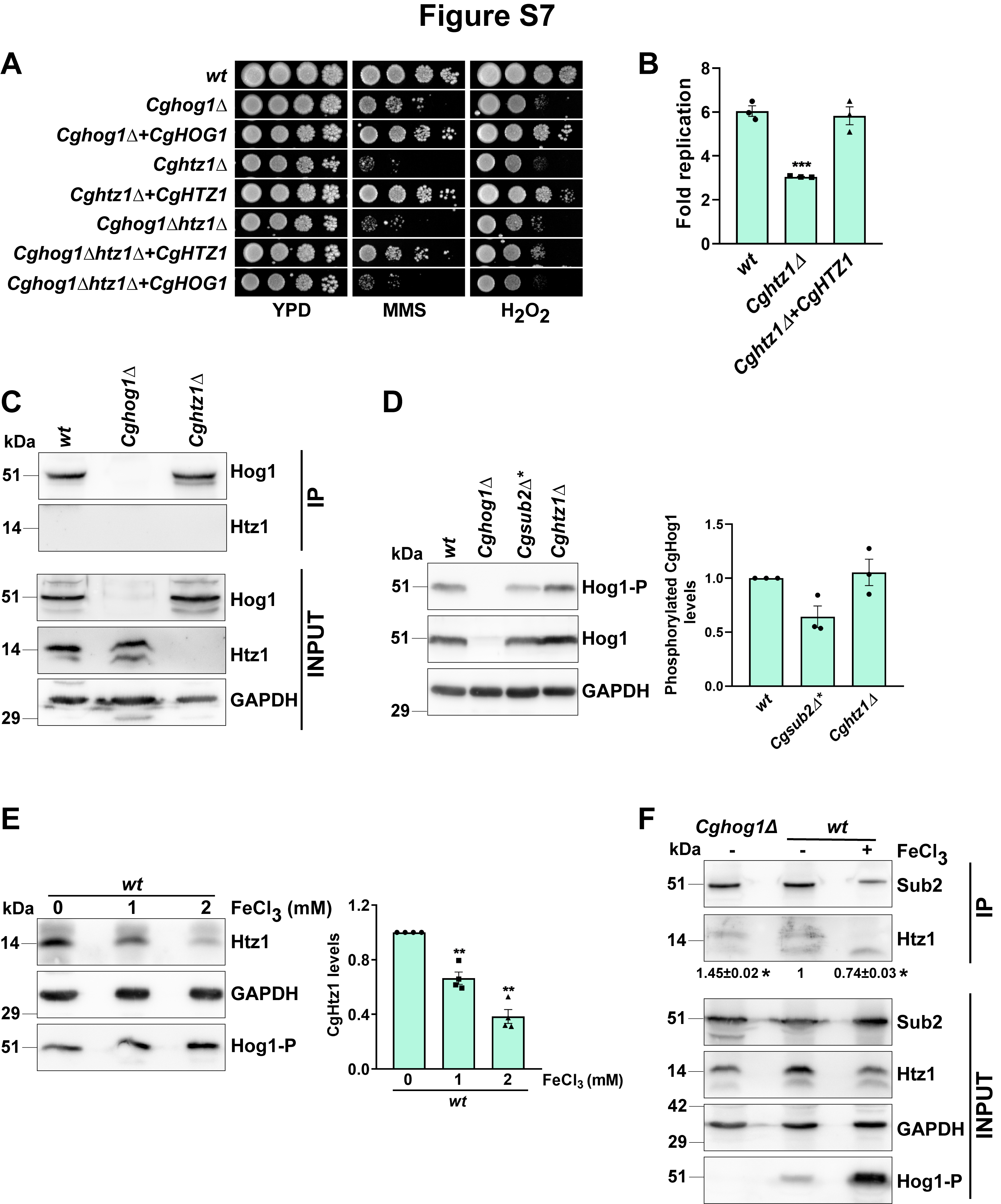

Supplement: S7 Fig — A. Serial dilution spotting analysis illustrating sensitivity of indicated strains to DNA damage [MMS (methyl methanesulfonate; 0.04%)] and oxidative [Hydrogen peroxide (H2O2; 20 mM)] stresses. Growth was recorded after 2–3 days of incubation at 30°C. wt, Cghog1Δ, Cghog1Δ+CgHOG1, Cghtz1Δ, Cghtz1Δ+CgHTZ1, Cghog1Δhtz1Δ, Cghog1Δhtz1Δ+CgHTZ1, Cghog1Δhtz1Δ+CgHOG1 strains correspond to YRK20, YRK964, YRK6290, YRK4392, YRK4737, YRK4390, YRK5123 and YRK6292 strains, respectively. B. Intracellular replication analysis. Human THP-1 monocytic cells were treated with PMA (phorbol 12-myristate 13-acetate; 16 nM) for 12 h, followed by recovery in the fresh RPMI medium for 12 h. Overnight YPD-medium-grown Cg strains were infected to differentiated THP-1 macrophages at MoI (multiplicity of infection) of 1:10. After 2 h incubation, non-internalized Cg cells were washed off with PBS, and the infection was continued for another 22 h. Infected macrophages were lysed in water at 2 h and 24 h post infection, and lysates were diluted in PBS and plated on YPD medium. After 1–2 days of incubation at 30°C, Cg colonies were counted, and the number was multiplied by the appropriate dilution factor. Fold replication (mean ± SEM; n = 3) represents the ratio of the number of intracellular Cg cells at 24 h to that at 2 h post infection for each strain. ***, p ≤ 0.001; unpaired two-tailed Student’s t test. wt, Cghtz1Δ and Cghtz1Δ+CgHTZ1 strains correspond to YRK20, YRK4392 and YRK4737 strains, respectively. C. CgHog1 does not interact with CgHtz1. Lysates (6 mg) of indicated, YNB medium-grown strains were incubated with anti-CgHog1 antibody for 12 h. Immunoprecipitated (IP) and cell lysate (Input) samples were resolved on 15% SDS-PAGE, followed by probing with anti-CgHog1, anti-Htz1 and anti-Gapdh antibodies. CgGapdh was used as loading control. wt, Cghog1Δ and Cghtz1Δ strains correspond to YRK20, YRK964 and YRK4392 strains, respectively. D. Immunoblot showing phosphorylated CgHog1 levels. Lysates [file pgen.1011281.s007.tif]

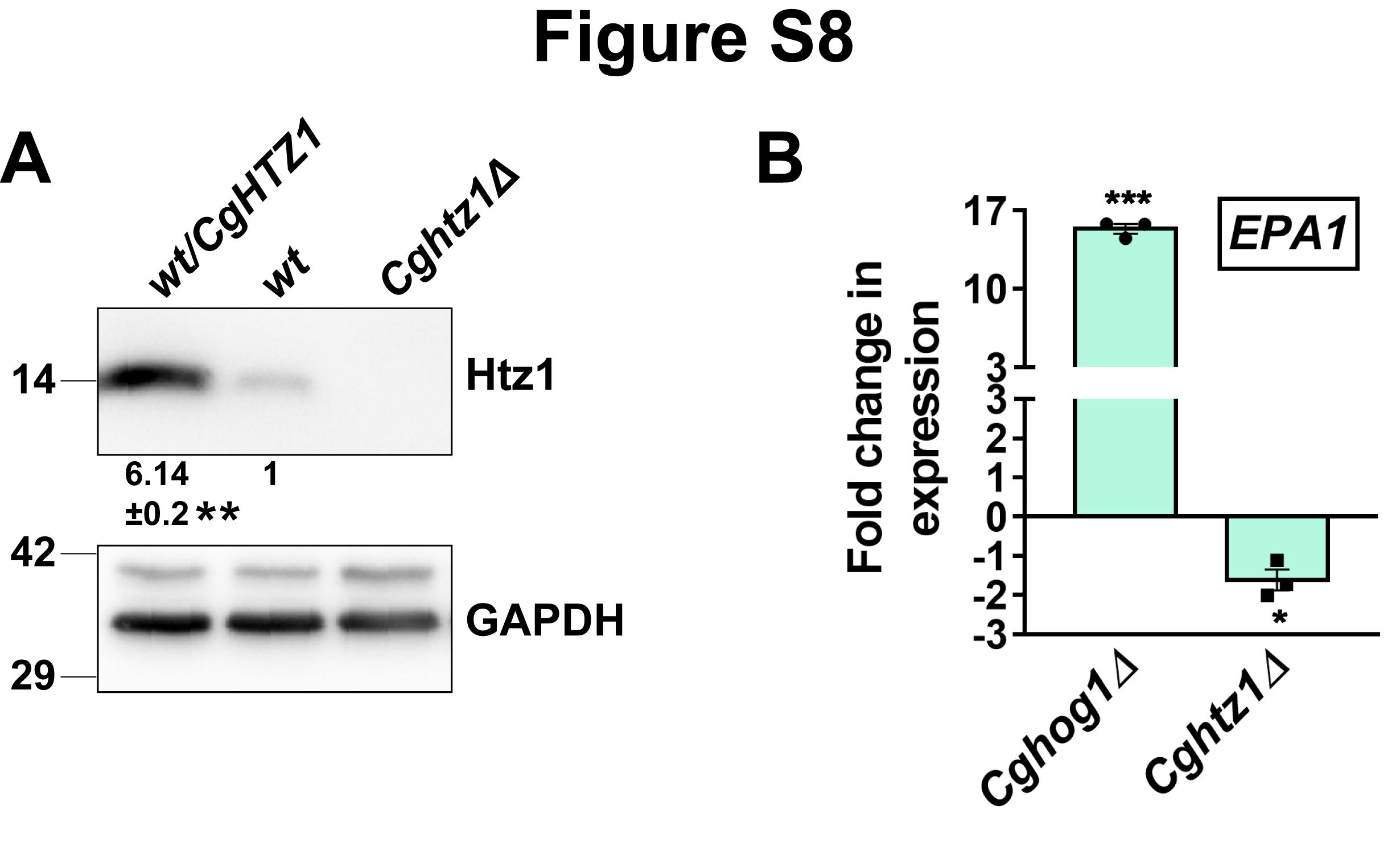

Supplement: S8 Fig — A. Immunoblot showing CgHtz1 levels upon CgHTZ1 overexpression. Overnight CAA medium-grown wt (YRK20), Cghtz1Δ (YRK4392) and wt cells overexpressing CgHTZ1 from PDC1 promoter (wt/HTZ1; YRK4729) were collected. Whole cell lysates were prepared and 100 μg samples were resolved on 15% SDS-PAGE, followed by immunoblotting with anti-Htz1 and anti-Gapdh antibodies. Data (mean ± SEM; n = 3) represent fold-increase in CgHtz1 levels in wt/HTZ1, compared to wt (taken as 1.0). **, p ≤ 0.01, paired two-tailed Student’s t-test. B. qRT-PCR-based EPA1 expression analysis. Data (mean ± SEM, n = 3) were normalized against CgACT1 mRNA control, and represent fold change in EPA1 transcript levels in log-phase Cghtz1Δ (YRK4392) and Cghog1Δ (taken as control; YRK964) cells, compared to log-phase wt (YRK20) cells (considered as 1.0). *p ≤ 0.05; ***, p ≤ 0.001, paired two-tailed Student’s t-test. (TIF) [file pgen.1011281.s008.tif]

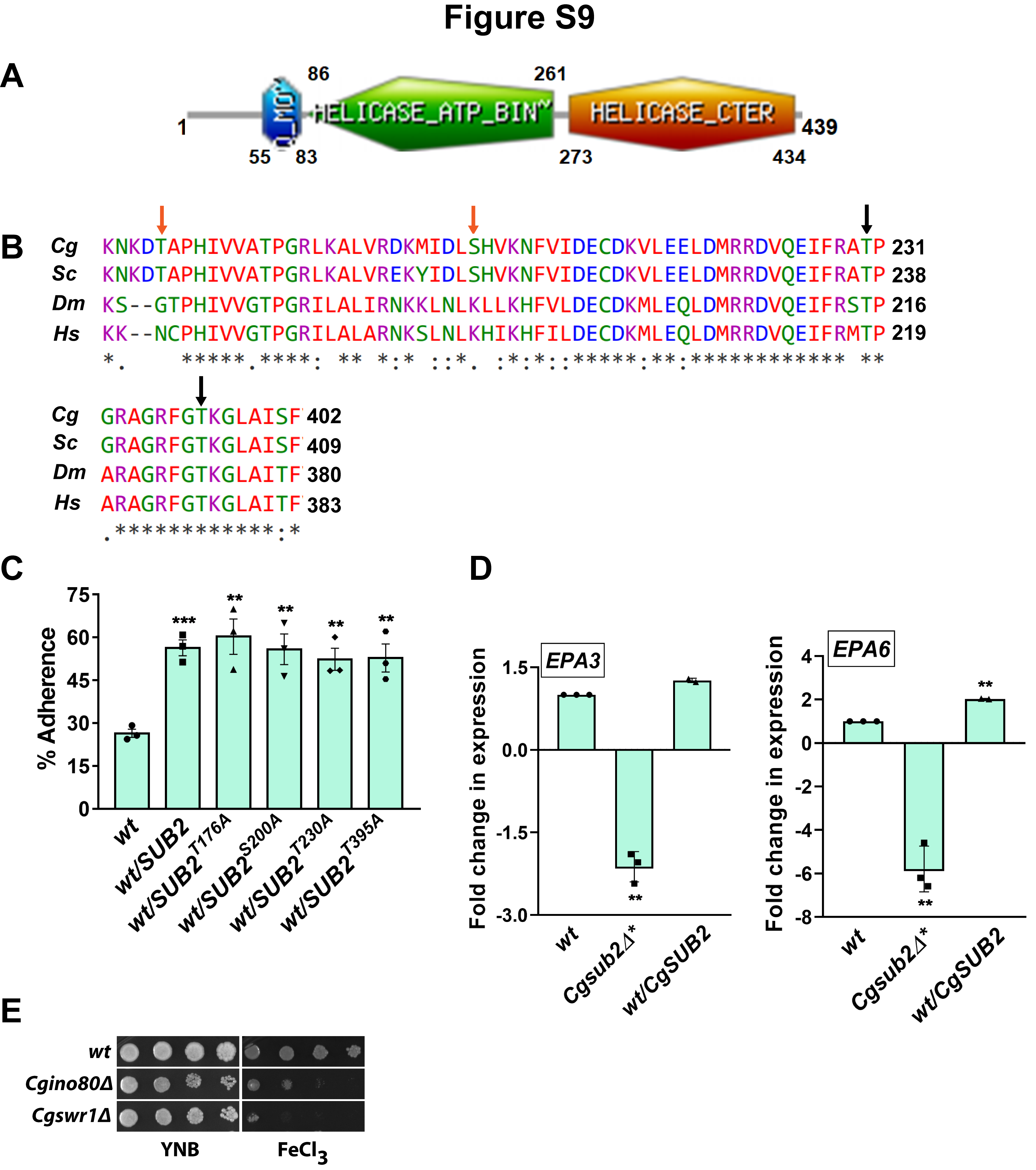

Supplement: S9 Fig — A. A schematic illustration of domains predicted in 439 amino acid-long CgSub2 protein using ExPASy PROSITE proteomics analysis server (https://prosite.expasy.org/prosite.html). Q_MOTIF (55–83 aa): DEAD-box RNA helicase Q motif, HELICASE_ATP_BIND_1 (86–261 aa): Superfamilies 1 and 2 helicase ATP-binding type-1 domain, and HELICASE_CTER (273–434 aa): Superfamilies 1 and 2 helicase C-terminal domain. The diagram is not drawn to scale. B. Multiple amino acid sequence alignment of Homo sapiens (Hs; DX39B), Saccharomyces cerevisiae (Sc; Sub2), Drosophila melanogaster (Dm; DX39B) and Candida glabrata (Cg; Sub2) Sub2 proteins showing conserved identified four phosphorylatable residues (Thr-176, Ser-200, Thr-230 and Thr-395) in CgSub2. Clustal Omega (https://www.ebi.ac.uk/Tools/msa/clustalo/) was used to align sequences retrieved from the Uniprot database (https://www.uniprot.org/) for each protein. The brown and black arrows mark the conserved residues between Cg and Sc Sub2, and Sub2 in all four organisms, respectively. C. Adherence analysis of S35-labelled wt cells, that were overexpressing CgSub2 variants with indicated serine and threonine residues mutated to alanine, to A-498 cells. Overexpression of both wild-type and mutated CgSub2 rendered Cg cells hyperadherent. Data represent mean ± SEM (n = 3). **p ≤ 0.01; ***p ≤ 0.001, unpaired two-tailed Student’s t-test. wt, wt/CgSUB2, wt/CgSUB2T176A, wt/CgSUB2S200A, wt/CgSUB2T230A and wt/CgSUB2T395A strains correspond to YRK20, YRK2803, YRK5167, YRK4640, YRK5121 and YRK5171 strains, respectively. D. qRT-PCR-based measurement of EPA3 and EPA6 transcript levels. wt (YRK20), Cgsub2Δ* (YRK3294) and wt/CgSUB2 (YRK2803) strains were grown to log-phase in YNB, methionine and cysteine-containing YNB and YNB medium, respectively. Total RNA was isolated, and 500 ng RNA was used for cDNA synthesis, followed by real-time quantitative PCR amplification. Transcript levels were quantified using the 2-ΔΔCt method. Data (mean ± SD, n = 2–3) [file pgen.1011281.s009.tif]
